# An Intraoperative Telemedicine Program to Improve Perioperative Quality Measures: The ACTFAST-3 Randomized Clinical Trial

**DOI:** 10.1001/jamanetworkopen.2023.32517

**Published:** 2023-09-22

**Authors:** Christopher R. King, Stephen Gregory, Bradley A. Fritz, Thaddeus P. Budelier, Arbi Ben Abdallah, Alex Kronzer, Daniel L. Helsten, Brian Torres, Sherry McKinnon, Shreya Goswami, Divya Mehta, Omokhaye Higo, Paul Kerby, Bernadette Henrichs, Troy S. Wildes, Mary C. Politi, Joanna Abraham, Michael S. Avidan, Thomas Kannampallil

**Affiliations:** 1 Department of Anesthesiology, Washington University School of Medicine in St Louis, St Louis, Missouri; 2 Department of Anesthesiology, University of Nebraska Medical Center, Omaha; 3 Institute for Informatics, Washington University School of Medicine in St Louis, St Louis, Missouri

## Abstract

**Importance:**

Telemedicine for clinical decision support has been adopted in many health care settings, but its utility in improving intraoperative care has not been assessed.

**Objective:**

To pilot the implementation of a real-time intraoperative telemedicine decision support program and evaluate whether it reduces postoperative hypothermia and hyperglycemia as well as other quality of care measures.

**Design, Setting, and Participants:**

This single-center pilot randomized clinical trial (Anesthesiology Control Tower–Feedback Alerts to Supplement Treatments [ACTFAST-3]) was conducted from April 3, 2017, to June 30, 2019, at a large academic medical center in the US. A total of 26 254 adult surgical patients were randomized to receive either usual intraoperative care (control group; n = 12 980) or usual care augmented by telemedicine decision support (intervention group; n = 13 274). Data were initially analyzed from April 22 to May 19, 2021, with updates in November 2022 and February 2023.

**Intervention:**

Patients received either usual care (medical direction from the anesthesia care team) or intraoperative anesthesia care monitored and augmented by decision support from the Anesthesiology Control Tower (ACT), a real-time, live telemedicine intervention. The ACT incorporated remote monitoring of operating rooms by a team of anesthesia clinicians with customized analysis software. The ACT reviewed alerts and electronic health record data to inform recommendations to operating room clinicians.

**Main Outcomes and Measures:**

The primary outcomes were avoidance of postoperative hypothermia (defined as the proportion of patients with a final recorded intraoperative core temperature >36 °C) and hyperglycemia (defined as the proportion of patients with diabetes who had a blood glucose level ≤180 mg/dL on arrival to the postanesthesia recovery area). Secondary outcomes included intraoperative hypotension, temperature monitoring, timely antibiotic redosing, intraoperative glucose evaluation and management, neuromuscular blockade documentation, ventilator management, and volatile anesthetic overuse.

**Results:**

Among 26 254 participants, 13 393 (51.0%) were female and 20 169 (76.8%) were White, with a median (IQR) age of 60 (47-69) years. There was no treatment effect on avoidance of hyperglycemia (7445 of 8676 patients [85.8%] in the intervention group vs 7559 of 8815 [85.8%] in the control group; rate ratio [RR], 1.00; 95% CI, 0.99-1.01) or hypothermia (7602 of 11 447 patients [66.4%] in the intervention group vs 7783 of 11 672 [66.7.%] in the control group; RR, 1.00; 95% CI, 0.97-1.02). Intraoperative glucose measurement was more common among patients with diabetes in the intervention group (RR, 1.07; 95% CI, 1.01-1.15), but other secondary outcomes were not significantly different.

**Conclusions and Relevance:**

In this randomized clinical trial, anesthesia care quality measures did not differ between groups, with high confidence in the findings. These results suggest that the intervention did not affect the targeted care practices. Further streamlining of clinical decision support and workflows may help the intraoperative telemedicine program achieve improvement in targeted clinical measures.

**Trial Registration:**

ClinicalTrials.gov Identifier: NCT02830126

## Key Points

**Question:**

Does a real-time intraoperative telemedicine program improve perioperative quality of care measures?

**Findings:**

In this randomized clinical trial of 26 254 patients having surgery at a single academic medical center, an intraoperative telemedicine decision support intervention did not significantly reduce postoperative hypothermia or hyperglycemia and did not significantly improve most perioperative quality of care measures. However, intraoperative glucose measurement in patients with diabetes was more common with the intervention.

**Meaning:**

These findings suggest that further streamlining of clinical decision support and workflows may help the intraoperative telemedicine program achieve improvement in targeted clinical measures.

## Teaser

This randomized clinical trial evaluates whether implementation of a real-time intraoperative telemedicine decision support program reduces postoperative hypothermia and hyperglycemia as well as other anesthesia care quality measures among adult surgical patients in the US.

## Introduction

The World Health Organization defines telemedicine as the provision of care services using communication technologies for diagnosis and treatment.^1^ Over the past decade, the use of telemedicine and clinical decision support has substantially increased.^2,3^ Telemedicine in the field of anesthesiology has emerged in preoperative assessments,^4,5,6^ remote intraoperative monitoring,^7,8,9,10^ and postoperative management.^11,12,13^ However, intraoperative telemedicine has been limited to monitoring of geographically remote operating rooms (ORs)^7,9,10^ and case studies of telementoring.^9,10,14^ Clinical decision support tools for intraoperative care have been found to improve some quality outcomes.^15^ However, findings from previous studies^16,17^ have emphasized the burden of alert fatigue, which reduces the benefit of decision support. Filtering of decision support alerts by telemedicine clinicians may mitigate alert fatigue and reduce oversights in care.

Adopting a user-centered design approach, we developed and implemented the Anesthesiology Control Tower (ACT), a real-time telemedicine decision support system.^18,19^ The ACT combines remote intraoperative monitoring with customized clinical decision support using the AlertWatch platform. The ACT clinicians monitor multiple ORs and review decision alerts to assess patient safety risks and offer preemptive recommendations to intraoperative anesthesiology clinicians. The present study, Anesthesiology Control Tower–Feedback Alerts to Supplement Treatments (ACTFAST-3), was a pilot randomized clinical trial (RCT) evaluating the feasibility of an efficacy trial of the ACT and its impact on 2 quality of care measures: postoperative hypothermia and hyperglycemia.^20^

## Methods

### Study Design, Setting, and Ethics

ACTFAST-3 was a single-center pilot superiority RCT conducted at Barnes-Jewish Hospital and Washington University School of Medicine in St Louis, Missouri, from April 3, 2017, to June 30, 2019. The data analysis was initially performed from April 22 to May 19, 2021, with updates in November 2022 and February 2023. The study site used a medical direction model for anesthesia care supervision, with no more than 4 (and usually 3) nurse anesthetists per anesthesiologist and usually 2 resident physicians per anesthesiologist. The trial protocol is provided in Supplement 1. The institutional review board of Washington University in St Louis granted approval of the study with a waiver of informed consent due to minimal risk to participants. This study followed the Consolidated Standards of Reporting Trials (CONSORT) reporting guideline for RCTs.^21^

### Study Population

**Figure 1.**
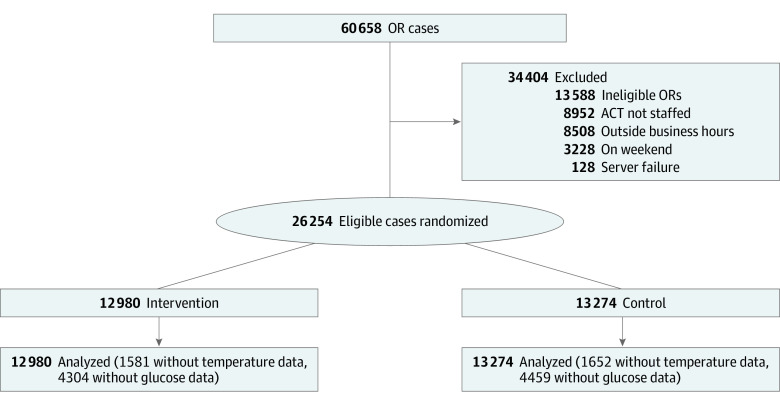
Study Flowchart The analysis included all patients randomized during the study with outcomes present. ACT indicates Anesthesiology Control Tower; and OR, operating room.

All patients 18 years or older who underwent surgery in 1 of 48 designated ORs were enrolled. During our institutional transition to the Epic electronic health record (EHR) from May 21 to September 10, 2018, data were excluded due to data quality and technical issues. Patients were excluded if greater than 50% of their case duration (anesthesia start to anesthesia stop time) occurred outside of the ACT staffed hours, which were typically from 7:00 am to 4:00 pm on Monday through Friday, with exclusions for technological failures and personnel shortages. The rationale for this exclusion was to focus on cases for which the ACT was able to make timely recommendations. For glycemic outcomes, patients without diabetes were excluded from analysis. Overall, 60 658 surgical procedures were performed during the study period. After exclusions (Figure 1), 26 254 patients (12 980 in the intervention group and 13 274 in the control group) were included in the analysis. Baseline patient characteristics were taken from the EHR; race, gender, and other characteristics were entered based on self-report. Race and ethnicity data were routinely collected for administrative purposes and subsequently taken from the EHR; they were not used in the study other than for reporting the demographic characteristics of the population.

### Randomization and Blinding

To reduce contamination effects and improve protocol adherence, ORs (rather than patients) were randomized 1:1 to the control or intervention group each day using centrally generated sequences. All patients in each OR for that day had the same treatment assignment. Operating room randomization was balanced on a 1:1 ratio for each day; however, because some ORs had no cases on some days, the allocation ratio varied randomly, with a mean of 1. Randomization was included in the ACT display interface. Patients received either usual intraoperative care comprising medical direction from the anesthesia care team (control group) or usual care augmented by telemedicine decision support from the ACT (intervention group).

Patients were blinded to group allocation. Operating room clinicians were unblinded if contacted by the ACT but were not aware of their allocation otherwise. Clinicians in the ACT monitored patients in the control group but did not contact intraoperative clinicians unless there was a patient safety issue necessitating immediate action, such as failure to deliver an anesthetic agent. There were no changes to OR staffing based on group allocation. Patients with another surgical procedure within 30 days after an index operation were analyzed according to their previous treatment assignment. Because the interface did not track individual-level previous randomizations, in subsequent procedures, patients received the treatment per the new OR day’s independent randomization. Surgical procedures received more than 30 days after an index operation were analyzed according to the new randomization. This strategy was chosen in the protocol to reduce contamination effects due to ACT-assisted management plans carried over to subsequent procedures. Sensitivity analyses excluded previous treatment assignments and used only the first operation in a 30-day window.

### Intervention

The ACT is a remote suite staffed by an anesthesiologist who is supported by a study coordinator trained in the study procedures, the decision support software interface, and communication tools. A total of 1 to 3 personnel, including nurse anesthetists, anesthesiology resident physicians, and nurse anesthetist trainees, also participated daily. The suite computers accessed the EHR and a customized version of the decision support platform. Although the protocol envisioned live waveform display and audio-video monitoring of ORs, these technological features were not available.

Descriptions of how the ACT interface was developed and adapted to clinician feedback to improve acceptability and usability have been published previously.^16,18,19^ The ACT protocols were refined with clinician stakeholders to maximize the value of recommendations. The ACT clinicians used a dashboard to prioritize review of high-acuity cases and cases with active alerts. The dashboard displayed patients in both the intervention and control groups. The decision support software display included physiological data, laboratory data, medical history, and other summaries as well as alerts.

Interactions between the ACT and OR clinicians evolved during the study.^16^ Initially, ACT clinicians filtered and communicated alerts to OR clinicians via phone calls because EHR-integrated messaging tools were not widely used by OR clinicians. The ACT shifted focus to delivering preemptive comprehensive assessments of patient risk and potential areas for risk mitigation in addition to communicating relevant alerts. The decision support software also evolved. For example, early in the pilot, a mean arterial pressure lower than 65 mm Hg would trigger an alert for hypotension, but later iterations included adjustable thresholds, tracked the overall duration of hypotension, and incorporated notes and recommendations based on that alert.

After the Epic EHR implementation (June 2018), the Epic In Basket messaging system was used for case reviews and nonurgent alerts. Phone calls were used for time-sensitive alerts. Clinicians in the ACT were encouraged to log OR communications and case reviews in the decision support software and to select an action or reason for silencing each alert. In the log, ACT staff rated the importance (referred to as *significance* in the log) of each issue, whether the OR team had noted or acted on the issue, and whether the ACT-OR communication changed medical management.

### Outcomes

The primary outcomes were avoidance of hypothermia, defined as the proportion of patients who had a final recorded intraoperative core temperature greater than 36 °C, and avoidance of hyperglycemia, defined as the proportion of patients with diabetes who had a blood glucose level of 180 mg/dL or lower on arrival to the postanesthesia recovery area. These outcomes were selected because they were viewed as clinically meaningful opportunities for quality improvement and were plausibly affected by ACT-OR communication.

Secondary outcomes included intraoperative hypotension, temperature monitoring, timely antibiotic redosing, intraoperative glucose evaluation and management, neuromuscular blockade monitoring, ventilator management, and volatile anesthetic overuse. Clinical outcomes included 30-day mortality, 30-day readmission, and postoperative acute kidney injury. Due to limited data availability, planned analysis of intraoperative awareness and surgical site infection outcomes were not conducted. Definitions of the primary, secondary, and clinical outcomes are provided in eTable 1 in Supplement 2. All outcomes were assessed using routinely captured EHR data.

### Sample Size Calculation

Sample size was calculated according to the previously published protocol.^22^ Because of intervention changes related to the Epic EHR transition, recruitment was extended to include 12 000 patients after the EHR transition.

### Statistical Analysis

Randomized patients were considered to have received the intervention and were included in the intention-to-treat analysis regardless of whether the ACT communicated with the OR. Continuous outcomes (time with low mean arterial pressure, time without antibiotics, and fresh gas flow) were analyzed using a linear generalized estimating equation model clustering on OR and day. Standard errors were calculated using the heteroskedasticity-consistent (HC) type 1 (HC1) estimator from sandwich package 3.0-0 in R software, version 4.0.4 (R Foundation for Statistical Computing).^23^ Acute kidney injury stage was analyzed using a proportional odds regression model with HC type 0 (HC0)–clustered SEs. All other outcomes were binary and analyzed with a Poisson regression model with HC0-clustered SEs to obtain rate ratios (RRs).^23^ Within each group of outcomes, *P* values were Holm adjusted for multiple testing. Confidence intervals were reported using Bonferroni-corrected α levels; this correction was not planned a priori but was compatible with the results post hoc. The threshold for statistical significance was 2-tailed *P* = .05. To visualize secular trends in intervention effects, we used regression analysis of each 3-month calendar segment and a linear generalized estimating equation model with the same SE approach used for other outcomes. The protocol planned comparison of patients in the intervention group with patients in the matched historical control group; however, the EHR transition halfway through the study made that comparison infeasible. A post hoc analysis was performed examining the type and frequency of alert communication to OR teams by the ACT clinicians. All data were analyzed using R software, version 4.0.4.

## Results

Among 26 254 patients included in the analysis, 13 393 (51.0%) identified as female, 12 852 (49.0%) as male, and 9 (0.03%) as other genders (not specified in the EHR), with a median (IQR) age of 60 (47-69) years. A total of 297 patients (1.1%) were Asian, 5327 (20.3%) were Black, 20 169 (76.8%) were White, and 461 (1.8%) were of other race (including American Indian or Alaska Native, multiple races, other race, unknown race, and declined to respond). There were 7681 clusters (OR days) in the intervention group and 7875 in the control group. Minimal differences in demographic characteristics, surgery type, functional status, and comorbidities were observed between the control group (n = 13 274) and the intervention group (n = 12 980) (Table 1). Overall, 65 clinicians in the ACT logged communication to ORs before the transition to the Epic EHR, and 87 logged communication to ORs after the transition to the Epic EHR.

**Table 1.** Patient Characteristics Stratified by Intervention

Characteristic	Patients, No./total No. (%)	
	Control group (n = 13 274)	Intervention group (n = 12 980)
Age, median (IQR), y	60 (47-69)	59 (47-69)
BMI, median (IQR)	29 (24-34)	29 (24-34)
Gender		
Female	6708/13 274 (50.5)	6685/12 980 (51.5)
Male	6562/13 274 (49.4)	6290/12 980 (48.5)
Other^a^	4/13 274 (0.03)	5/12 980 (0.04)
Race		
Asian	150/13 274 (1.1)	147/12 980 (1.1)
Black	2704/13 274 (20.4)	2623/12 980 (20.2)
White	10 197/13 274 (76.8)	9972/12 980 (76.8)
Other^b^	223/13 274 (1.7)	238/12 980 (1.8)
Surgery type		
Orthopedic	1509/13 274 (11.4)	1478/12 980 (11.4)
Cardiac and thoracic	1467/13 274 (11.1)	1357/12 980 (10.5)
Gynecological	1395/13 274 (10.5)	1392/12 980 (10.7)
Urological	1402/13 274 (10.6)	1287/12 980 (9.9)
General	972/13 274 (7.3)	998/12 980 (7.7)
Neurological	897/13 274 (6.8)	904/12 980 (7.0)
Otolaryngological	886/13 274 (6.7)	821/12 980 (6.3)
Vascular	793/13 274 (6.0)	802/12 980 (6.2)
Gastroenterological	469/13 274 (3.5)	503/12 980 (3.9)
Colorectal	436/13 274 (3.3)	430/12 980 (3.3)
Transplant	389/13 274 (2.9)	367/12 980 (2.8)
Hepatobiliary	344/13 274 (2.6)	377/12 980 (2.9)
Plastics	175/13 274 (1.3)	154/12 980 (1.2)
Other	2140/13 274 (16.1)	2110/12 980 (16.3)
ASA physical status classification		
1	550/11 932 (4.6)	558/11 703 (4.8)
2	4661/11 932 (39.1)	4546/11 703 (38.8)
3	5379/11 932 (45.1)	5378/11 703 (46.0)
4	1312/11 932 (11.0)	1188/11 703 (10.2)
5	30/11 932 (0.3)	33/11 703 (0.3)
Barthel Index <100^c^	1414/13 274 (10.7)	1479/12 980 (11.4)
Coronary artery disease	1664/12 154 (13.7)	1583/11 913 (13.3)
Congestive heart failure	1312/12 154 (10.8)	1233/11 913 (10.4)
Atrial fibrillation	1167/12 154 (9.6)	1085/11 913 (9.1)
Peripheral arterial disease	1033/12 154 (8.5)	950/11 913 (8.0)
Diabetes	2909/10 296 (28.3)	2824/10 091 (28.0)
Cirrhosis	262/12 154 (2.2)	256/11 913 (2.1)
Functional capacity <4 METs	3264/10 702 (30.5)	3291/10 596 (31.1)
Hypertension	6397/12 154 (52.6)	6260/11 913 (52.5)
COPD or asthma	2430/12 154 (20.0)	2379/11 913 (20.0)
End-stage kidney disease	567/12 529 (4.5)	573/12 274 (4.7)
Cerebrovascular disease	882/12 154 (7.3)	766/11 913 (6.4)
Current cancer	2627/12 154 (21.6)	2597/11 913 (21.8)

Abbreviations: ASA, American Society of Anesthesiologists; BMI, body mass index (calculated as weight in kilograms divided by height in meters squared); COPD, chronic obstructive pulmonary disease; MET, metabolic equivalent of task.

^a^ Specific genders included in the *other* category were not specified in the electronic health record.

^b^ Includes electronic health record entries of American Indian or Alaska Native, multiple races, other race, unknown race, and declined to respond.

^c^ The Barthel Index measures independence in activities of daily living, with scores ranging from 0 (severe dependence in all domains) to 100 (complete independence in assessed domains).

As a pilot trial, demonstrating the ability to deliver the intervention was a main aim of this study. Gaps in staffing and software problems decreased over time. In 2017 (April 3 through December 31), the ACT was operational on 156 of 192 weekdays. From October 1, 2018, through June 30, 2019, 182 of 192 weekdays were staffed. The number of staffed weekdays per month is shown in eFigure 1A in Supplement 2; temporal patterns in the number of alerts and the number of OR communications are shown in eFigure1B and eFigure 1C, respectively, in Supplement 2. The number of alerts, alert-related OR communications, and case reviews by randomization status and EHR are shown in eTable 2 in Supplement 2.

Separation between the intervention and control ORs was excellent. Overall, the ACT contacted 1636 of 12 980 intervention ORs (12.6%) and 99 of 13 274 control ORs (0.7%). In the pre–Epic EHR period, 877 of 5808 intervention ORs (15.1%) and 40 of 6065 control ORs (0.7%) were contacted; in the Epic EHR period, 759 of 7172 intervention ORs (10.6%) and 59 of 7209 control ORs (0.8%) were contacted (eTable 2 in Supplement 2). Documented reasons for contacting a control OR included patient safety emergencies (n = 13) and researcher error (n = 6).

In the pre–Epic EHR period, the ACT was characterized by contacts for alerts (eTable 2 in Supplement 2); in the Epic EHR period, the ACT was characterized by a split between case review recommendations and alerts. The number of alerts increased substantially after the EHR transition (from 18 769 in the pre–Epic EHR period to 107 376 in the Epic EHR period) due to expanded alert definitions and a higher frequency of data updates. The most common alerts were related to hemodynamics and postoperative nausea prophylaxis, and few alerts were related to the primary outcomes of avoidance of postoperative hypothermia or hyperglycemia (eTable 3 in Supplement 2). The ACT staff indicated that the issue being communicated was “significant” in 791 cases and that their communication affected management in 634 of these cases (80.2%). Overall, the ACT reported that management was affected in 603 of 12 980 cases (4.6%) in the intervention group and 31 of 13 274 in the control group (0.2%) (eTable 2 in Supplement 2).

Results for primary, secondary, and exploratory clinical outcomes are shown in Table 2. For the primary outcomes, there was no significant difference in the avoidance of postoperative hypothermia (7602 of 11 447 patients [66.4%] in the intervention group vs 7783 of 11 672 [66.7%] in the control group; RR, 1.00; 95% CI, 0.97-1.02) or postoperative hyperglycemia (7445 of 8676 patients [85.8%] in the intervention group vs 7559 of 8815 [85.8%] in the control group; RR, 1.00; 95% CI, 0.99-1.01) between the control and intervention groups. For secondary outcomes, there was a significant increase in the incidence of appropriate intraoperative glucose measurement among patients with diabetes in the intervention group (RR, 1.07; 95% CI, 1.01-1.15; Holm-corrected *P* = .02). Among the clinical outcomes, a nonsignificant increase in postoperative 30-day readmission was observed in the intervention group (RR, 1.17; 95% CI, 1.00-1.37; Holm-corrected *P* = .07). Surgical site infections were removed from the analysis plan but did not differ between groups (372 of 10 178 patients [3.7%] in the intervention group vs 416 of 10 397 [4.0%] in the control group; RR, 0.91; 95% CI, 0.79-1.05).

**Table 2.** Primary, Secondary, and Clinical Outcomes

Outcome^a^	Patients, No./total No. (%)		Coefficient (95% CI)^b^	*P* value^c^
	Intervention group (n = 12 980)	Control group (n = 13 274)		
**Primary**				
No postoperative hypothermia	7602/11 447 (66.4)	7783/11 672 (66.7)	1.00 (0.97 to 1.02)	>.99
No postoperative hyperglycemia	7445/8676 (85.8)	7559/8815 (85.8)	1.00 (0.99 to 1.01)	>.99
**Secondary**				
Intraoperative glucose measurement	1346/1962 (68.7)	1274/1996 (63.8)	1.07 (1.01 to 1.15)	.02
Intraoperative low MAP, mean (SD)	7 (17)	7 (18)	−0.27 (−0.88 to 0.33)	>.99
Temperature monitoring	8612/9255 (93.1)	8766/9467 (92.6)	1.00 (0.99 to 1.02)	>.99
No missed antibiotics	12 743/12 980 (98.2)	13 031/13 274 (98.2)	1.00 (1.00 to 1.00)	>.99
Insulin per clinical guidelines	644/1007 (64.0)	721/1079 (66.8)	0.96 (0.88 to 1.04)	>.99
Neuromuscular monitoring documented	4649/6591 (70.5)	4581/6656 (68.8)	1.02 (0.99 to 1.06)	.22
Appropriate tidal volume	5053/5405 (93.5)	5066/5435 (93.2)	1.00 (0.99 to 1.02)	>.99
Fresh gas flow, mean (SD), L/min	3 (1)	3 (1)	0.01 (−0.05 to 0.07)	>.99
**Clinical**				
AKI stage^d^				
0	11 459/12 189 (94.0)	11 664/12 436 (93.8)	0.96 (0.83 to 1.11)	>.99
1	553/12 189 (4.5)	583/12 436 (4.7)		
2	77/12 189 (0.6)	74/12 436 (0.6)		
3	100/12 189 (0.8)	115/12 436 (0.9)		
30-d mortality	200/12 980 (1.5)	238/13 274 (1.8)	0.86 (0.67 to 1.11)	.45
30-d readmission	583/12 980 (4.5)	511/13 274 (3.8)	1.17 (1.00 to 1.37)	.07
Delirium^e^	216/616 (35.1)	218/643 (33.9)	1.03 (0.84 to 1.27)	>.99
Respiratory failure^e^	235/11 318 (2.1)	235/11 464 (2.0)	1.01 (0.79 to 1.30)	>.99
Incident atrial fibrillation^e^	297/11 720 (2.5)	349/11 921 (2.9)	0.87 (0.70 to 1.06)	.33

Abbreviations: AKI, acute kidney injury; MAP, mean arterial pressure.

^a^ Outcome definitions are provided in eTable 1 in Supplement 2.

^b^ For binary outcomes (reported as numbers with percentages) and ordinal outcomes (reported as medians with IQRs), the coefficient represents the rate ratio. For continuous outcomes (reported as means with SDs), the coefficient represents the regression coefficient. The 95% CIs were derived using a generalized estimating equation clustered on operating room and day.

^c^ *P* values were Holm corrected for each group of outcomes (2 primary, 8 secondary, and 6 clinical tests).

^d^ AKI stage (based on criteria from Kidney Disease: Improving Global Outcomes).

^e^ Delirium, respiratory failure, and atrial fibrillation categories exclude patients who had those conditions preoperatively.

**Figure 2.**
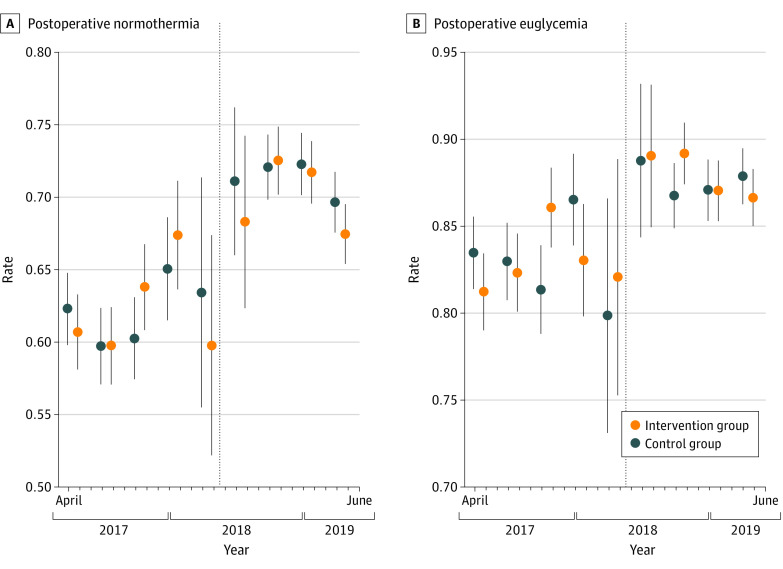
Time Course of Primary Outcomes The intervention group received usual care plus the live telemedicine intervention; the control group received usual care only. Error bars represent 95% CIs derived from a linear probability model with cluster-robust SEs. The dotted line represents transition to the Epic electronic health record.

There was a gradual improvement in postoperative hypothermia but no obvious pattern in intervention effects (Figure 2). Segmented regression results for secondary and clinical outcomes are shown in eFigure 2 in Supplement 2; some sharp changes at the EHR transition likely reflected changes in data capture and documentation as well as other changes in patient characteristics and clinical practices over time. Descriptive statistics and treatment effects, excluding subsequent surgical procedures within 30 days of an index operation or analysis with the assigned treatment (excluding previous assigned treatments), are shown in eTables 4 to 7 in Supplement 2. There was no difference in the frequency of multiple cases per patient between groups (2336 of 12 179 cases [19.2%] in the intervention group vs 2410 of 12 457 [19.3%] in the control group; RR, 0.99; 95% CI, 0.94-1.05), and no conclusions were substantially different.

The protocol planned an analysis limited to ORs with at least 1 alert. However, based on the high frequency of alerts (eTable 2 in Supplement 2), this analysis was not conducted.

## Discussion

In this pilot RCT with 26 254 patients, compared with usual care, a telemedicine decision support intervention for OR anesthesia clinicians did not significantly change 2 quality of care outcomes: avoidance of postoperative hypothermia and hyperglycemia. Although there was a small but statistically significant increase in intraoperative glucose measurement in the intervention group, there was no difference in the frequency of treatment for hyperglycemia, which may explain the lack of difference in postoperative hyperglycemia. To our knowledge, this is the first RCT of an intraoperative telemedicine intervention. In contrast to previous studies,^7,8,9^ our study incorporated a novel telemedicine platform that combined real-time data and clinical decision support with a streamlined approach for collaborative decision-making with OR clinicians, allowing clinicians to monitor many ORs simultaneously.

There are several potential reasons for the lack of an intention-to-treat effect with tight confidence bounds on these quality of care outcomes. First, there were likely substantial contamination and Hawthorne effects. All anesthesiology clinicians at the study site were aware that the ACT was monitoring all ORs and were aware of the process measures being studied. This awareness was reinforced by intermittent messages from the ACT about study patients, likely leading to spillover changes in practice as the clinicians looked for the same issues about which they tended to receive messages from the ACT. That is, the study estimand did not account for shared quality improvements due to ACT implementation and surveillance. For example, if the ACT reinforced institutional guidelines on blood glucose management for a clinician on 1 day, on a subsequent day during which that clinician was randomized to the control OR, they were likely to have remembered and followed those guidelines. Anesthesiologists could also supervise ORs in different treatment groups on the same day, increasing contamination.

Second, the overall adherence to each measure at baseline was high, making statistically significant improvements difficult to achieve. Independent hospital-based quality improvement efforts targeting these measures probably mitigated the benefit of the ACT. For example, improved EHR alerts about antibiotic redosing reduced the need for ACT notifications. The unmodified decision support application with default alerts was available to intraoperative staff during the study, which could have decreased the effect of the ACT. However, our anecdotal impression was that few non-ACT clinicians used the software. Third, the rate of recommendations was lower than expected. The ACT contacted only 12.6% of intervention ORs and reported changing management in only 4.6% of intervention ORs (eTable 2 in Supplement 2). The ACT clinicians relied on in-depth medical record review in addition to decision support, limiting their ability, time, and resources to find actionable recommendations. Recommendations for the primary outcomes depended minimally on detailed data, meaning that there were few mechanisms through which decision support could affect those outcomes. Relatively few alerts were related to the primary outcomes (eTable 3 in Supplement 2).

In future studies, the high labor input per actionable recommendation could be improved using algorithms to better filter patients and issues for review. The ACT used alerts based on simple rules (eg, hypotension present). With the rapidly expanding value of machine learning, algorithms have been developed, validated, and implemented to track estimated postoperative mortality,^24^ postoperative complications,^25^ transfusion,^26^ and surgical progress and duration.^27,28^ These algorithms could improve the value and value per time spent of ACT review by identifying patients who may have greater benefit from anticipatory planning and by increasing timely risk mitigation recommendations. The study site is another factor potentially reducing the observed effect; an academic medical center during business hours provides a high baseline level of monitoring. The intervention could have been more useful in times and places with more thinly stretched resources, such as night shifts.

Despite the lack of an impact on outcomes, the ACTFAST-3 pilot RCT provided a template for real-time intraoperative telemedicine support. Our approach incorporated 3 key aspects: an integrated data pipeline with granular EHR data, alerts for clinical decision support, and expert clinicians assessing the alerts and engaging in collaborative decision-making. There are several opportunities for improving intraoperative telemedicine support based on our experience. Refining the alert systems to recognize likely low-relevance alarms is a future direction. A recent interview and focus group study^16^ found that OR clinicians appreciate telemedicine’s role in improving patient safety and providing a new perspective for review of perioperative data. Although the study occurred over 2 years and had a large sample, we consider it a pilot rather than a definitive trial because the intervention and its delivery evolved during the study, including workflow changes during an EHR transition period.

### Strengths and Limitations

This study has several strengths. It is a large and rigorously conducted pragmatic trial using mixed methods to maximize the intervention.^18^ An academic-private partnership allowed rapid development of substantial infrastructure, including adapting to a major EHR transition. By incorporating educational and quality improvement, a larger number of clinicians were able to work within the ACT and improve its relevance.

This study also has limitations. Practices within the ACT and the decision support software evolved over the course of the trial. However, OR and ACT clinicians viewed these changes favorably, and there was no evidence of temporal heterogeneity. The trial was paused during an EHR transition period, which came with a learning curve for the ACT clinicians that included changing alerts, changing data availability, and new communication modalities. It is difficult to extrapolate the results beyond an academic center. There are multiple factors, including contamination and Hawthorne effects, which may have caused the study to underestimate the benefit of the ACT. A cluster-randomized study would be optimal to estimate the impact of the ACT on outcomes.

## Conclusions

This large single-center pilot RCT found that support from an intraoperative telemedicine center augmented by real-time clinical decision support did not affect intraoperative quality of care measures. These findings suggest that further streamlining of clinical decision support and workflows may help the intraoperative telemedicine program achieve improvement in targeted clinical measures.

## Notes

Supplement 1.
Trial Protocol

Supplement 2. **eTable 1.** Outcome Definitions **eTable 2.** ACT Contact Activity **eTable 3.** Common Alerts **eTable 4.** Descriptive Statistics Excluding Surgeries Within 30 Days of an Index Operation **eTable 5.** Treatment Effects Excluding Surgeries Within 30 Days of an Index Operation **eTable 6.** Descriptive Statistics Stratified by Intervention, Ignoring Prior Treatment Assignments in Subsequent Surgeries **eTable 7.** Treatment Effects, Ignoring Prior Treatment Assignments in Subsequent Surgeries **eFigure 1A.** Number of Days per Month With Included Cases (ACT Staffed, Randomization Operational, Outcomes Measured) **eFigure 1B.** Mean Number of Alerts per Case Over Time **eFigure 1C.** Number of Contacts per Case Over Time **eFigure 2.** Secondary and Clinical Outcomes Over Time

Supplement 3. **Nonauthor Collaborators.** Members of the ACTFAST Study Group

Supplement 4.
Data Sharing Statement
