# Supplementary material for: An Intraoperative Telemedicine Program to Improve Perioperative Quality Measures: The ACTFAST-3 Randomized Clinical Trial
Source: JAMA Netw Open. 2023 Sep 22;6(9):e2332517. doi: 10.1001/jamanetworkopen.2023.32517 (PMC10517374; doi:10.1001/jamanetworkopen.2023.32517)
Supplement: Supplement 1. — Trial Protocol [file jamanetwopen-e2332517-s001.pdf]

ACTFAST

PI: Michael Avidan
IRB ID #: 201603038

Project Details

1. Demographics

1.1 Project Title:
Anesthesiology Control Tower: Feedback Alerts to Supplement Treatments (ACTFAST)

1.2 Short Title (required):
ACTFAST

1.3 Project is primarily:
Biomedical

1.3.a Does this study require review under ICH-GCP?
No

1.4 Type of Study:
Other Interventional

1.4.a Is your research study one in which one or more human subjects are prospectively assigned to one or more interventions (which may include placebo or other control) to evaluate the effects of those interventions on health-related biomedical or behavioral outcomes (NIH clinical trial definition).
No

1.5 Select how you plan to obtain consent:
- No consent process (waiver of consent)

2. Source(s) of Support

2.1 Source(s) of Support

Type/Source	Grant Title	Name of PI on Grant	Status
Departmental			
Federal Agency<br>Agency for Healthcare Research & Quality (DHHS)	Anesthesiology Control Tower: feedback Alerts to supplement Treatment	Michael S. Avidan	AWARDED

Attachment Name	Category	Version	Date Attached
ApplicationFINAL_Redacted.pdf	Grant from funding source or private foundation/association	1	04/03/17

3. Research Team

3.1 Principal Investigator

Name	E-mail	Title	Sch
Michael Avidan	avidanm@wustl.edu	S & R Brown Prof of Anesthesiology	Sch

3.2 Team Members
Research Team Members

Role	Name	Role Desc	Student	Email	Title	School	Department	Con
PI	Michael Avidan, MD		No	avidanm@wustl.edu	S & R Brown Prof of Anesthesiology	School of Medicine	Anesthesiology - Non Clinical Operations - Non Clinical Administration	Ye
	Joanna Abraham, PHD		No	joannaa@wustl.edu	Assoc Prof of Anesthesiology	School of Medicine	Anesthesiology - Non Clinical Operations - Research	N
	Amrita Aranake-Chrisinger, BA, MD			aranakea@wustl.edu	Asst Prof of Anesthesiology	School of Medicine	Anesthesiology - Non Clinical Operations -	N

							Non Clinical Administration	
	Umeshkumar Athiraman, MD		No	uathira@wustl.edu	Assoc Prof of Anesthesiology	School of Medicine	Anesthesiology - Non Clinical Operations - Non Clinical Administration	N
	Arbi Ben Abdallah, DES, PHD			aba@wustl.edu	Assoc Prof of Anesthesiology	School of Medicine	Anesthesiology - Non Clinical Operations - Non Clinical Administration	N
	Mara Bollini, BA, BSN, MHA			bollinim@wustl.edu	Performance Improvement Consul	School of Medicine	Anesthesiology - Non Clinical Operations - Non Clinical Administration	N
	Thaddeus Budelier, MD, MSF			thaddeus.budelier@wustl.edu	Program Manager	School of Medicine	Anesthesiology - Non Clinical Operations - Research	N
	Yixin Chen, PHD			ychen25@wustl.edu	Professor	McKelvey School of Engineering	Engineering - Computer Science and Engineering	N
	Bradley Fritz, MD			bafritz@wustl.edu	Asst Prof of Anesthesiology	School of Medicine	Anesthesiology - Non Clinical Operations - Research	N
	Stephen Gregory, MD			shgregory@wustl.edu	Assoc Prof of Anesthesiology	School of Medicine	Anesthesiology - Non Clinical Operations - Non Clinical Administration	N
	Daniel Helsten, MD			helstend@wustl.edu	Assoc Prof of Anesthesiology	School of Medicine	Anesthesiology - Non Clinical Operations - Non Clinical Administration	N
	Bernadette Henrichs, PHD			henrichsb@wustl.edu	Prof of Anesthesiology	School of Medicine	Anesthesiology - Non Clinical Operations - Non Clinical Administration	N
	Thomas Kannampallil, PHD		No	thomas.k@wustl.edu	Assoc Prof of Anesthesiology	School of Medicine	Anesthesiology - Non Clinical Operations - Research	N
	Christopher King, MD, PHD			christopherking@wustl.edu	Asst Prof of Anesthesiology	School of Medicine	Anesthesiology - Non Clinical Operations - Research	N
	Lecheng Kong, MS		No	jerry.kong@wustl.edu		GRADUATE ARTS AND SCIENCES	Computer Science and Engineering	N
	Alexander Kronzer, BA			akronzer@wustl.edu	Medical Informaticist II	School of Medicine	Anesthesiology - Non Clinical Operations - Non Clinical Administration	N
	Dingwen Li, MS		No	dingwenli@wustl.edu	Graduate Research Assistant	McKelvey School of Engineering	Engineering - Computer Science and Engineering	N
	Hao Liu, BS		No	liuhao@wustl.edu	Graduate Research Assistant	McKelvey School of Engineering	Engineering - Computer Science and Engineering	N
	Sherry McKinnon, BS, MS, CCRP			smckinnon@wustl.edu	Mgr Div of Clinical Research	School of Medicine	Anesthesiology - Non Clinical Operations - Research	N
	Alicia Meng, BA		No	aliciameng@wustl.edu	Clinical Research Coordinator I	School of Medicine	Anesthesiology - Non Clinical Operations - Research	N
	Melissa Milbrandt, High School		No	melissa.milbrandt@wustl.edu	Business Director	School of Medicine	Anesthesiology - Non Clinical Operations -	N

							Non Clinical Administration	
	Arianna Montes de Oca, MD		No	a.montesdeoca@wustl.edu	Resident	School of Medicine	Anesthesiology - Non Clinical Operations - Non Clinical Administration	N
	Teresa Murray-Torres, MD			murrayt@wustl.edu	Asst Prof of Anesthesiology	School of Medicine	Anesthesiology - Non Clinical Operations - Non Clinical Administration	N
	Sarah Perez, MSN, CRNA, MS			perezs@wustl.edu	Nurse Anesthetist	School of Medicine	Anesthesiology - Non Clinical Operations - Non Clinical Administration	N
	Andrea Reidy, MD, 30071728		No	a.reidy@wustl.edu	Asst Prof of Anesthesiology	School of Medicine	Anesthesiology - Non Clinical Operations - Non Clinical Administration	N
	Pratyush Sontha, BA		No	pratyushsontha@wustl.edu	•Visiting Program Trainee	School of Medicine	Anesthesiology - Non Clinical Operations - Research	N
	Brian Torres, MS, DNP, CRNA		No	btorres@wustl.edu	Nurse Anesthetist	School of Medicine	Anesthesiology - Non Clinical Operations - Non Clinical Administration	N
	Sandhya Tripathi, PHD		No	sandhyat@wustl.edu	Postdoc Research Associate	School of Medicine	Anesthesiology - Non Clinical Operations - Research	N
	Troy Wildes, MD			wildest@wustl.edu	Assoc Prof of Anesthesiology	School of Medicine	Anesthesiology - Non Clinical Operations - Non Clinical Administration	N
	Rachel Wolfe, PharmD			Rachel.Wolfe@bjc.org	SUPV, CLINICAL PHARMACY SPEC		Pharmacy (18001002)	N
	Bing Xue, BS, MS		No	xuebing@wustl.edu	Graduate Research Assistant	McKelvey School of Engineering	Engineering - Computer Science and Engineering	N

Team Member Financial Interest

Name	Financial Interests
Michael Avidan, MD	none
Joanna Abraham, PHD	none
Amrita Aranake-Chrisinger, BA, MD	none
Umeshkumar Athiraman, MD	none
Arbi Ben Abdallah, DES, PHD	none
Mara Bollini, BA, BSN, MHA	none
Thaddeus Budelier, MD, MSF	none
Yixin Chen, PHD	none
Bradley Fritz, MD	none
Stephen Gregory, MD	none
Daniel Helsten, MD	none
Bernadette Henrichs, PHD	none
Thomas Kannampallil, PHD	none
Christopher King, MD, PHD	none
Lecheng Kong, MS	none
Alexander Kronzer, BA	none
Dingwen Li, MS	none
Hao Liu, BS	none
Sherry McKinnon, BS, MS, CCRP	none
Alicia Meng, BA	none
Melissa Milbrandt, High School	none
Arianna Montes de Oca, MD	none
Teresa Murray-Torres, MD	none
Sarah Perez, MSN, CRNA, MS	none
Andrea Reidy, MD, 30071728	none
Pratyush Sontha, BA	none
Brian Torres, MS, DNP, CRNA	none

Sandhya Tripathi, PHD	none
Troy Wildes, MD	none
Rachel Wolfe, PharmD	none
Bing Xue, BS, MS	none

4. Other Institutional Reviews/Requirements

4.1 Do any of the objectives of this study involve the diagnosis, prevention, screening, evaluation, treatment or support of cancer patients?
No

4.2 Are more than 30% of the patients involved in this study likely to have an active cancer diagnosis?
No

4.3 Will any subject be asked to undergo a radiation therapy procedure (including external beam therapy, brachytherapy, or radiopharmaceutical therapy)?
No

4.4 Does your study involve the administration of non therapeutic radiopharmaceuticals (radioactive drugs) for research purposes?
No

4.5 Will any participant be asked to undergo any of the following:

- a standard radiology procedure involving ionizing radiation (includes X-rays, fluoroscopy, DEXA, CT)
OR
- a standard nuclear medicine examination with FDA-approved radioactive drugs (including bone scans, radionuclide ventriculogram (RVG or MUGA), myocardial perfusion imaging, FDG-PET)
- **DO NOT include a nuclear medicine examination performed with the investigational radioactive drug(s) listed above in Question 4.4.**
- DO NOT include MRI or ultrasound

No

4.6 Will the study involve any of the following activity **PROSPECTIVELY** at WUSM or any BJC hospitals, even if subjects or their insurance will not be billed for the item or service, and regardless of the study funding source (including studies with departmental or no funding)?

- Procedures, tests, examinations, hospitalizations, use of Pathology, Laboratory, Cardiology, or Radiology services, use of clinic facilities or clinical equipment, or any patient care services, including services conducted in the Clinical Research Unit; or
- Physician services or services provided by non-physicians who are credentialed to bill (ARNPs, Physician Assistants, etc.)

Yes

4.6.a Will any of the prospective activities **EVER** use clinic facilities or clinical equipment **OUTSIDE** of a Designated Research Area? A Designated Research Area is one which does not schedule, order, or result/document via Epic. No insurance information is collected from subjects in a Designated Research Area.

Examples of Designated Research Areas:

- CTRU (Clinical & Translational Research Unit)
- CLCS (Core Laboratory for Clinical Studies)
- ID-CRU (Infectious Disease-Clinical Research Unit)
- NCRU (Neuroclinical Research Unit)
- ADRC (Alzheimer's Disease Research Center)
- CCIR (Center for Clinical Imaging) *Studies that request or require a professional radiology read be done and documented in Epic, this question must be answered "Yes".*

No

4.7 Does this study involve administration of recombinant or synthetic nucleic acids (gene therapy or mRNA vaccines) or microorganisms?
No

4.8 Does this study involve the use of human embryonic stem cells or human induced pluripotent stem cells?
No

4.9 Does this study involve research in which a human embryo or embryos are destroyed, discarded, or knowingly subjected to risk of injury or death greater than that allowed for research on fetuses in utero?
No

4.12 Will a Certificate of confidentiality be used for this research?
No

4.13 Does this project need to be registered on ClinicalTrials.gov?
Yes

4.13.a Who is the Responsible Party for registering this study in ClinicalTrials.gov?
Principal Investigator

4.14 Title that should appear in Epic (and will be visible in the patient medical record):

ACTFAST

4.15 Select one person from the study team that should appear in Epic as the contact person for this study:
Sherry McKinnon

4.16 Do you want to request that an ordering tool be built for your study in Epic?
No

4.17 Would you like to submit a request for the Epic team to consider your study for the use of BPA (Best Practice Advisory) in Epic?
No

4.18 Would you like to submit a request for the Epic team to build your questionnaires in Epic for the purposes of recruitment?
No

4.19 Will any external monitors require access to this study in Epic?
No

4.21 Mark all that apply to your study:

1. Protocol

1.1 Is there a separate, written protocol that will be submitted in addition to this form? (Note: a grant application is not considered to be a protocol)
No

1.2 Select up to three key words below that best describe this research study:
- Anesthesiology

1.3 Provide a short summary/abstract of the purpose and procedures of the study proposed in this IRB application.

- DO NOT include information on studies not proposed in this application.
- Use LAY terminology only. This must be easily understandable by IRB community members and nonscientists.
- DO NOT cut and paste technical abstracts from source of support applications that may not be understood by a general audience.

The purpose of this study is to implement an Anesthesiology Control Tower (ACT). This pilot study will employ the existing information technology infrastructure at Barnes Jewish Hospital to create a remote monitoring and alerting system for the operating rooms. Similar to “telemedicine” methods employed in critical care, the ACT will support operating room clinicians in adhering to best-practice principles.

The proposed study will logically build on our previous work and utilize the already established infrastructure and resources of the SATISFY-SOS registry study, the intra-operative electronic medical record and the AlertWatch® evidence-based alerting system. In conceptualizing this pilot study, as well as a larger follow-up trial focused on clinical outcomes, we have been mindful of challenges put forward to investigators by the National Institutes of Health, which seek to support low-cost, pragmatic, patient-centered randomized controlled trials (82). Specifically in relation to ACTFAST, (1) the highly developed, specialized IT infrastructure limits the investment required for embarking on this study; (2) the study will be conducted entirely within the context of routine clinical care, negating the need for dedicated trial-related visits; (3) access to registries with granular data on complications and patient-reported outcomes obviates the need for a new and costly infrastructure to track patient outcomes; (4) inclusion of large numbers of broadly representative patients in this study will be highly efficient with a waiver of informed consent (82). The design for this pilot proof-of-concept study will be a randomized clinical effectiveness trial of two years duration. It will include a 3 month pre-intervention period during which time the ACT will be staffed but no alerts will be sent. This will allow for the training of controllers, refinement of alerts, and optimization of processes for obtaining and filtering information from diverse electronic sources. This will also allow for the determination of baseline event rates for the outcomes of interest. Following this period, the trial will begin and run for 21 months. On a daily basis during the study period (weekdays from 7am to 5pm), each operating room at Barnes-Jewish Hospital where anesthesiology services are provided will be eligible for randomization, which will occur daily at the level of the operating room using computer-generated assignment. The experimental arm will receive the additional support of the Anesthesiology Control Tower in the form of control-tower alerts to the attending anesthesiologist that complement the AlertWatch® system. Some of these alerts will use video conference calling for communication between the clinician in the control tower and the clinicians in the operating room. The outcomes of interest in this pilot study will be the usability and usefulness of the ACT, clinician adherence to recommendations for monitoring, documentation and therapeutic interventions, key physiologic variables such as temperature and blood pressure, and patient outcomes including duration of postoperative hospital stay, incidence of postoperative morbidity (myocardial infarction, surgical site infection), functional recovery, and postoperative quality of life. All of the alerts included in this study will be chosen because they follow best intraoperative management practice and are in line with national metrics for quality and safety (83-85).

Outcome data will be collected on a group level to see if when the control tower provided information and intervention followed.

1.4 Specify your research question(s), study aims or hypotheses:
Specific Aim 1: To determine the usefulness and usability of an Anesthesiology Control Tower (ACT) for the operating suite.
- We hypothesize that the ACT will be usable and will be perceived as useful by the anesthesia team.
Specific Aim 2: To determine whether the ACT improves:
a. Clinicians’ compliance with standards of practice for treatment, documentation, and monitoring.
b. Surrogate measures of patient outcomes.
- We hypothesize that the ACT will result in (a) better clinician compliance (e.g., timely administration of antibiotics, rigorous documentation, and regular blood pressure and neuromuscular monitoring) and (b) improvement of key physiological parameters (e.g., temperature and blood pressure).
Specific Aim 3: To explore whether the ACT improves postoperative patient outcomes.
- We hypothesize that when the ACT provides feedback to clinicians, there will be a trend towards improved patient outcomes (e.g., fewer readmissions, improved quality of life) in this pilot trial.

1.5 Background and significance and/or Preliminary studies related to this project:

A.1. Opportunity for information and communication technology to improve perioperative care

Most of the >200 million people (1) who undergo elective surgery and other procedures around the world every year choose to do so because they believe that the surgery will cure a condition or improve their quality of life.(2) However, in recent years research conducted at Barnes-Jewish Hospital (BJH) and around the world has revealed that the incidence of adverse outcomes after surgery is disturbingly high. Following in-patient surgeries, the estimated 30-day mortality is between 1% and 5% (3-11), and by 90 days to 1 year postoperatively, between 5% and 10% of these patients have died (5, 6, 9, 12). Furthermore, about 10% to 20% of surgical patients experience major complications, such as heart attacks, unremitting pain, infections and blood clots, in the weeks to months following their procedures (7, 10, 11, 13). We are currently embarked on a major quality improvement initiative at BJH, called SATISFY-SOS (Systematic Assessment and Targeted Improvement of Services Following Yearling Surgical Outcomes Surveys - NCT02032030), in which we are systematically tracking the health and well-being of surgical patients postoperatively. This is a unique initiative enrolling 10,000 patients a year that will allow us to gauge outcomes that are important to patients, such as duration of debility, postoperative quality of life, and the timeline for return to work and to full functionality. Additionally, we have partnered locally with the American College of Surgeons' National Surgical Quality Improvement Program (NSQIP) and the Society of Thoracic Surgery (STS) database in order to enhance the granularity of the measured outcomes. Given the increased availability of information and communication technology at our hospital, and our current ability to track patients' postoperative trajectories, we believe that the next priority in our quality improvement agenda should be to leverage information and communication technology to implement perioperative evidence-based practices that positively impact patients' outcomes.

A.2. Information and communication technology can aid implementation of evidence-based care

The disturbing frequency of medical errors and patient harm has been well publicized (14). Unfortunately, increases in public and clinician recognition of errors, and the resulting movements in research and health care policy, have failed to decrease the rate at which harm occurs (15). In addition to directly committing errors, clinicians continue to contribute to harm by failing to implement evidence-based standards of care. This failure has been documented repeatedly and in a variety of settings (16, 17); whether in internal medicine (18) or critical care (19), clinicians do not follow even well established guidelines. Possibly of most concern is the lack of clinician awareness of these failures; many clinicians believe that they are in compliance with guidelines when in reality they are not (20). Overall, it is clear that the pursuit of quality in the healthcare system still has much to achieve and many barriers to overcome. Appropriate integration of information technology (IT) to provide decision support and meaningful alerts could potentially overcome barriers to the implementation of evidence-based care (21-23).

A.3. IT-driven decision support can impact outcomes

Despite major advancements in the safety of anesthetic techniques and therapeutics, the risk of patient morbidity and mortality related to surgery persists. Some of this risk is unavoidable and is either inherent to the nature of the surgical procedure itself, or is attributable to a characteristic of the patient that is not in an immediate way modifiable (10, 24, 25). There are, however, factors that do fall under the control of the anesthetic team that have been shown to affect the patient's immediate and long term health (26-29), including the appropriate management of hyperglycemia (30), proper administration of prophylactic antibiotics (31, 32), maintenance of normothermia (33), the avoidance of excessively large tidal volumes with mechanical ventilation (34, 35), administration of sufficient anesthesia (36-39), and appropriate thresholds for blood transfusions (40, 41). IT-driven decision support has been shown to optimize management of these factors, leading to improvement in physiological measures, such as blood pressure stability (42) and glucose control (43, 44) We have demonstrated that an IT-based solution can improve appropriate repeat dosing of antibiotics during surgery, although there remains opportunity for further improvement. We have also shown through large pragmatic clinical trials that IT-driven decision support can impact clinically relevant outcomes, such as preventing patients from experiencing intraoperative awareness (37). More generally, there is emerging evidence that perioperative management practices guided by IT solutions are likely to have lasting and long-term effects on patient outcomes (12). Leaders in perioperative quality improvement and patient safety must therefore develop pragmatic, technology-driven methods to implement such practices into the routine management paradigm.

A.4. Need for empowering and unobtrusive IT-based solutions

Information overload is a major barrier to implementation of evidence-based practice in the operating room. Anesthesiologists, like all medical practitioners, have multiple known limitations in their cognitive and decision making abilities (45-47). There is a limited amount of data that physicians can process per unit of time(48), and the mismatch between human decision making ability and excess information is a source of medical errors (49). In the realm of anesthesiology, the operating room environment is a source of numerous stimuli that can at times overwhelm a single clinician, who is often inundated with alarms and alerts. Practitioners may see alarms as frequently as every 3 minutes—even more frequently during induction of anesthesia and emergence from anesthesia—and while the majority of alarms might appear to be clinically irrelevant, a small percentage do require immediate intervention (50). Less experienced practitioners are likely to have the most difficulty and require the most assistance in these circumstances. However even the performance of senior physicians can suffer from the cognitive workload involved in patient care, particularly when supervising inexperienced trainees (51). Further information overload through intrusive or overwhelming IT-based decision support is likely to be unappreciated and even counter-productive. Given the known limits of human cognitive abilities, there is therefore a pressing need for empowering and unobtrusive perioperative information-technology based systems that support critical decision-making without inundating the clinician with unfiltered and often irrelevant information (52, 53).

A.5. Innovative information-technology based solution for the OR

The advancement of information technology and clinician decision support systems is being championed in the demand for quality in health care (54, 55), and in recent years, anesthesiology departments have increasingly embraced this development. Eight years ago, only 16% of the academic anesthesiology departments in the country employed anesthesia information management systems in their ORs (56); now, they are used in more than 75% of anesthesia departments in the United States(57). Since their inception, the functionality of anesthesia information management systems has increased remarkably, and they have been used to improve documentation compliance (58), to increase adherence to guidelines for prophylactic perioperative antibiotics (59, 60) and post-operative nausea and vomiting prophylaxis (61), and also to assist in the intraoperative management of hypotension and hypertension (42). Unfortunately, adherence has been shown to drop off over time (61). Data from remote electronic monitoring and support studies in the ICU strongly suggest that the addition of "telemedicine" may be a method for improving and maintaining adherence to established best practices (62, 63). On the strength of these compelling data, there has been widespread adoption of remote monitoring centers for intensive care units that provide back up to clinicians on the ground (64). Surprisingly, this control center model has not been evaluated for intraoperative care despite the fact that major perturbations in physiology (e.g., hemorrhage, shock, hypothermia, arrhythmias, hypoxemia, acid-base derangements) are probably more commonly encountered in the operating room than in the intensive care unit. By the time such derangements are recognized in the OR and rescue measures are initiated, damage to organ systems may have already occurred. There is therefore a gap in the field with an urgent need to assess the potential utility of a remote IT-based control center for the OR to anticipate and prevent injury and to improve quality of care generally. The insights yielded by the proposed ACTFAST (Anesthesiology Control Tower: Feedback Alerts to Supplement Treatments) pilot study will be highly informative to future research seeking to clarify the potential for health IT to promote evidence-based perioperative care.

B. Theoretical Framework for this Health IT Intervention Project
B.1 Anesthesiology Control Tower (ACT) Concept
The key innovative concept underlying this proposed health IT intervention project is that an Anesthesiology Control Tower (analogous to an air traffic control tower) can improve medical care for patients in the OR. Similar IT interventions have been developed in the critical care environment (65-67). These systems provide integrated information at the point of care, thus facilitating individualized care at the bedside and serving as platforms for broad quality improvement initiatives (68, 69). In the OR, this approach is now made possible at our hospital by technological advances in clinical monitoring and display systems. Such advances include real-time access to the hospital's electronic information systems, treatment guidelines, and protocols for care, as well as the availability of AlertWatch® (Ann Arbor, MI) in all ORs. Importantly, the perioperative electronic medical record, all hospital information systems, and the AlertWatch® dashboard are accessible remotely on any secure device with Internet connection (see figures 1a and 1b). This provides the opportunity to monitor patients remotely, and when necessary to institute ancillary support and even timely rescue interventions. AlertWatch® is an FDA cleared (Kl3O4Ol) patient monitoring and alerting system that provides integrated information similar to those systems developed in critical care. The crucial feature of AlertWatch® that is being used in this study is the availability of information via a secure Internet connection to clinicians at any physical location in the world. This allows for the creation of the remote Anesthesiology Control Tower (ACT). Just as an air traffic control tower monitors each aircraft and delivers additional information and alerts to the pilot and co-pilot, this ACT will engage with each team of anesthesia clinicians in a similar fashion to assist them in providing safe, effective, and efficient care for their patients. Also, just as a pilot may not be in the cockpit with the co-pilot for the entire flight, supervising anesthesiologists are not continuously present in the ORs. In our analogy, these physicians will be alerted by the supplementary ACT when undesired events are occurring or have the potential to occur, enabling the supervisors ("pilots") to return and provide appropriate support to the clinicians ("co-pilots") in the OR. The processing of electronic data by physician "controllers" in the ACT is a key component of the ACT, as it is expected that they will filter and prioritize information in order to improve the quality of alerts and to limit false alarms to the clinicians in the OR. This conceptualization of the ACT could allow it to decrease the burden from false and intrusive alarms in the OR, and instead provide an empowering and unobtrusive IT-based decision support solution to clinicians.

B.2. Overcoming challenges in the implementation of a health IT solution
The ACT concept provides a practical and innovative solution to the challenge of implementing evidence-based quality improvement in the operating room. It is abundantly clear that the deficiency of evidence-based practice in medicine is not due to lack of evidence; dozens of research studies are published every day and over 800,000 studies are available in the Cochrane Register of Controlled Trials (70). A gap between knowledge and performance is highlighted by the fact that increased awareness of areas of poor performance in patient care unfortunately may not lead to improvements in this care, as recently shown for hospitals enrolled in NSQIP. Participation in this quality-reporting program was not associated with any improved surgical outcomes in either of two rigorous studies (71, 72). In response to the known deficiency of evidence-based care, clinical decision support systems and similar technologies have been recommended by a number of organizations (23, 54, 73) as a method to help align clinical decisions with best practices, through the provision of relevant, evidence-based information and information management tools at the point of care (22). Encouragement is provided by a recent proof-of-concept study demonstrating that implementation of the AlertWatch® infrastructure in the ORs at the University of Michigan led to desirable intraoperative glycemic management behavior (44). Still, clinicians may fail to make use of decision support resources (74), and benefits from computerized decision support fade with time (18). Research performed in the intensive care field has repeatedly demonstrated that the addition of real-time, in-person (75, 76) or near-real time (77) prompting of clinicians improves adherence to best practices and furthermore improves patient outcomes. The ACT concept will provide similar real-time prompting based on data contained in AlertWatch® and other electronic health information systems, thus maximizing the potential to achieve individualized, evidence-based patient management in the operating suite.

B.3. Behavioral and organizational considerations
Genuine interest from key decision makers is an established facilitator to implementing new information technology in the healthcare setting (78, 79). With the strong support of the departmental chair, the residency director, and the department's anesthesiologists and residents, the ACT concept is primed for success. Fifteen attending anesthesiologists have already volunteered to spend fifteen days each a year as the "controller" in the ACT as part of their non-clinical allotment, and the residency curriculum will be revised to allow each senior resident the opportunity to gain invaluable experience as the "deputy controller" in the ACT. Given the continual, substantial increase in requirements for surgical procedures, a projected shortage of anesthesiologists, and financial constraints in healthcare, it will be infeasible for anesthesiologists to provide the level of supervision that is currently standard in the United States (e.g. one anesthesiologist for every one to four operating rooms) (80). However, current education of anesthesiologists largely emphasizes management of individual patients. There is little training on multi-tasking, on the supervision of anesthesia care teams, and on process management; yet anesthesiologists are already expected to be skilled in all of these areas, and the expectation will become increasingly manifest. Future anesthesiologists will be required not only to be competent in the development and implementation of individual anesthetic plans for patients who are presenting with ever-more complex medical conditions (81), but also in the management and direction of several concurrent plans delivered by residents or non-physician anesthetic clinicians. Resident participation in the staffing of the ACT will be a formative clinical experience that introduces trainees to the ever-expanding leadership roles that they will need to fill following graduation.

B.4. Likelihood for ACTFAST to generate insight
The ACTFAST study will make use of the highly granular perioperative electronic medical record to generate insight into surrogate perioperative measures (e.g., temperature, glucose, blood pressure management) that are likely to be associated with meaningful patient outcomes. Furthermore, this study will benefit from the extensive collaborative infrastructure that has been established over the last three years under the auspices of the Institute of Quality Improvement, Research and Informatics at Washington University. Despite the vast number of inpatient surgeries that take place in the United States (approximately 50 million each year), it is currently not routine to systematically track patients' postoperative outcomes after hospital discharge. At Washington University, approximately 25% of surgical patients have participated through structured questionnaires in the SATISFY-SOS initiative since 2012, through which we are tracking surgical outcomes up to one year postoperatively using electronic health information and patient-reported outcome measures. Patient reported outcomes tracked include health-related quality of life (Veteran's Rand-12), medical complications, falls, functional status (Barthel Index), cognition (PROMIS Cognition), pain, and intraoperative awareness (Modified Brice Interview). In order to augment outcomes tracked, we are enriching the SATISFY-SOS registry with extensive data from the electronic health record as well as from the local NSQIP and STS databases. The ACTFAST pilot study will therefore be well positioned to explore the association between intraoperative management and patient outcomes up to one year postoperatively, with minimal additional cost to the proposed project.

1.6 Literature cited/references (if attaching a grant enter N/A):
1. Weiser TG, Regenbogen SE, Thompson KD, Haynes AB, Lipsitz SR, Berry WR, Gawande AA. An estimation of the global volume of surgery: a modelling strategy based on available data. Lancet. 2008;372(9633):139-44. doi: 10.1016/S0140-6736(08)60878-8. PubMed PMID: 18582931.

2. Oresanya LB, Lyons WL, Finlayson E. Preoperative assessment of the older patient: a narrative review. JAMA : the journal of the American Medical Association. 2014;311(20):2110-20. doi: 10.1001/jama.2014.4573. PubMed PMID: 24867014.
3. Pearse RM, Moreno RP, Bauer P, Pelosi P, Metnitz P, Spies C, Vallet B, Vincent JL, Hoeft A, Rhodes A, European Surgical Outcomes Study group for the Trials groups of the European Society of Intensive Care M, the European Society of A. Mortality after surgery in Europe: a 7 day cohort study. Lancet. 2012;380(9847):1059-65. doi: 10.1016/S0140-6736(12)61148-9. PubMed PMID: 22998715; PMCID: 3493988.
4. Noordzij PG, Poldermans D, Schouten O, Bax JJ, Schreiner FA, Boersma E. Postoperative mortality in The Netherlands: a population-based analysis of surgery-specific risk in adults. Anesthesiology. 2010;112(5):1105-15. PubMed PMID: 20418691.
5. Kertai MD, Pal N, Palanca BJ, Lin N, Searleman SA, Zhang L, Burnside BA, Finkel KJ, Avidan MS, Group BUS. Association of perioperative risk factors and cumulative duration of low bispectral index with intermediate-term mortality after cardiac surgery in the B-Unaware Trial. Anesthesiology. 2010;112(5):1116-27. Epub 2010/04/27. doi: 10.1097/ALN.0b013e3181d5e0a3. PubMed PMID: 20418692.
6. Kertai MD, Palanca BJ, Pal N, Burnside BA, Zhang L, Sadiq F, Finkel KJ, Avidan MS, Group BUS. Bispectral index monitoring, duration of bispectral index below 45, patient risk factors, and intermediate-term mortality after noncardiac surgery in the B-Unaware Trial. Anesthesiology. 2011;114(3):545-56. Epub 2011/02/05. doi: 10.1097/ALN.0b013e31820c2b57. PubMed PMID: 21293252.
7. Healey MA, Shackford SR, Osler TM, Rogers FB, Burns E. Complications in surgical patients. Archives of surgery. 2002;137(5):611-7; discussion 7-8. PubMed PMID: 11982478.
8. Finlayson EV, Birkmeyer JD. Operative mortality with elective surgery in older adults. Effective clinical practice : ECP. 2001;4(4):172-7. PubMed PMID: 11525104.
9. Visser BC, Keegan H, Martin M, Wren SM. Death after colectomy: it's later than we think. Archives of surgery (Chicago, Ill : 1960). 2009;144(11):1021-7. Epub 2009/11/18. doi: 10.1001/archsurg.2009.197. PubMed PMID: 19917938.
10. Turrentine FE, Wang H, Simpson VB, Jones RS. Surgical risk factors, morbidity, and mortality in elderly patients. Journal of the American College of Surgeons. 2006;203(6):865-77. doi: 10.1016/j.jamcollsurg.2006.08.026. PubMed PMID: 17116555.
11. Hamel MB, Henderson WG, Khuri SF, Daley J. Surgical outcomes for patients aged 80 and older: morbidity and mortality from major noncardiac surgery. Journal of the American Geriatrics Society. 2005;53(3):424-9. doi: 10.1111/j.1532-5415.2005.53159.x. PubMed PMID: 15743284.
12. Monk TG, Saini V, Weldon BC, Sigl JC. Anesthetic management and one-year mortality after noncardiac surgery. Anesthesia and analgesia. 2005;100(1):4-10. Epub 2004/12/24. doi: 10.1213/01.ane.0000147519.82841.5e. PubMed PMID: 15616043.
13. Story DA, Leslie K, Myles PS, Fink M, Poustie SJ, Forbes A, Yap S, Beavis V, Kerridge R, Reason Investigators A, New Zealand College of Anaesthetists Trials G. Complications and mortality in older surgical patients in Australia and New Zealand (the REASON study): a multicentre, prospective, observational study. Anaesthesia. 2010;65(10):1022-30. doi: 10.1111/j.1365-2044.2010.06478.x. PubMed PMID: 20731639.
14. Institute of Medicine Committee on Quality of Health Care in A. In: Kohn LT, Corrigan JM, Donaldson MS, editors. To Err is Human: Building a Safer Health System. Washington (DC): National Academies Press (US)
Copyright 2000 by the National Academy of Sciences. All rights reserved.; 2000.
15. Landrigan CP, Parry GJ, Bones CB, Hackbarth AD, Goldmann DA, Sharek PJ. Temporal trends in rates of patient harm resulting from medical care. The New England journal of medicine. 2010;363(22):2124-34. Epub 2010/11/26. doi: 10.1056/NEJMsa1004404. PubMed PMID: 21105794.
16. Cabana MD, Rand CS, Powe NR, Wu AW, Wilson MH, Abboud PA, Rubin HR. Why don't physicians follow clinical practice guidelines? A framework for improvement. Jama. 1999;282(15):1458-65. Epub 1999/10/27. PubMed PMID: 10535437.
17. Grol R, Grimshaw J. From best evidence to best practice: effective implementation of change in patients' care. Lancet. 2003;362(9391):1225-30. Epub 2003/10/22. doi: 10.1016/s0140-6736(03)14546-1. PubMed PMID: 14568747.
18. Demakis JG, Beauchamp C, Cull WL, Denwood R, Eisen SA, Lofgren R, Nichol K, Woolliscroft J, Henderson WG. Improving residents' compliance with standards of ambulatory care: results from the VA Cooperative Study on Computerized Reminders. Jama. 2000;284(11):1411-6. Epub 2000/09/16. PubMed PMID: 10989404.
19. Needham DM, Colantuoni E, Mendez-Tellez PA, Dinglas VD, Sevransky JE, Dennison Himmelfarb CR, Desai SV, Shanholtz C, Brower RG, Pronovost PJ. Lung protective mechanical ventilation and two year survival in patients with acute lung injury: prospective cohort study. BMJ (Clinical research ed). 2012;344:e2124. Epub 2012/04/12. doi: 10.1136/bmj.e2124. PubMed PMID: 22491953; PMCID: Pmc3320566.
20. Steinman MA, Fischer MA, Shlipak MG, Bosworth HB, Oddone EZ, Hoffman BB, Goldstein MK. Clinician awareness of adherence to hypertension guidelines. The American journal of medicine. 2004;117(10):747-54. Epub 2004/11/16. doi: 10.1016/j.amjmed.2004.03.035. PubMed PMID: 15541324.
21. El-Kareh R, Hasan O, Schiff GD. Use of health information technology to reduce diagnostic errors. BMJ quality & safety. 2013;22 Suppl 2:ii40-ii51. Epub 2013/07/16. doi: 10.1136/bmjqs-2013-001884. PubMed PMID: 23852973; PMCID: PMC3786650.
22. Lobach D, Sanders GD, Bright TJ, Wong A, Dhurjati R, Bristow E, Bastian L, Coeytaux R, Samsa G, Hasselblad V, Williams JW, Wing L, Musty M, Kendrick AS. Enabling health care decisionmaking through clinical decision support and knowledge management. Evidence report/technology assessment. 2012(203):1-784. Epub 2012/11/07. PubMed PMID: 23126650.
23. Bates DW, Kuperman GJ, Wang S, Gandhi T, Kittler A, Volk L, Spurr C, Khorasani R, Tanasijevic M, Middleton B. Ten commandments for effective clinical decision support: making the practice of evidence-based medicine a reality. Journal of the American Medical Informatics Association : JAMIA. 2003;10(6):523-30. Epub 2003/08/20. doi: 10.1197/jamia.M1370. PubMed PMID: 12925543; PMCID: Pmc264429.
24. Lee TH, Marcantonio ER, Mangione CM, Thomas EJ, Polanczyk CA, Cook EF, Sugarbaker DJ, Donaldson MC, Poss R, Ho KK, Ludwig LE, Pedan A, Goldman L. Derivation and prospective validation of a simple index for prediction of cardiac risk of major noncardiac surgery. Circulation. 1999;100(10):1043-9. Epub 1999/09/08. PubMed PMID: 10477528.
25. Bilimoria KY, Liu Y, Paruch JL, Zhou L, Kmiecik TE, Ko CY, Cohen ME. Development and evaluation of the universal ACS NSQIP surgical risk calculator: a decision aid and informed consent tool for patients and surgeons. Journal of the American College of Surgeons. 2013;217(5):833-42.e1-3. Epub 2013/09/24. doi: 10.1016/j.jamcollsurg.2013.07.385. PubMed PMID: 24055383; PMCID: Pmc3805776.
26. Kheterpal S, Tremper KK, Englesbe MJ, O'Reilly M, Shanks AM, Fetterman DM, Rosenberg AL, Swartz RD. Predictors of postoperative acute renal failure after noncardiac surgery in patients with previously normal renal function. Anesthesiology. 2007;107(6):892-902. Epub 2007/11/29. doi: 10.1097/01.anes.0000290588.29668.38. PubMed PMID: 18043057.
27. Aronson S, Stafford-Smith M, Phillips-Bute B, Shaw A, Gaca J, Newman M. Intraoperative systolic blood pressure variability predicts 30-day mortality in aortocoronary bypass surgery patients. Anesthesiology. 2010;113(2):305-12. Epub 2010/06/24. doi: 10.1097/ALN.0b013e3181e07ee9. PubMed PMID: 20571360.
28. Biccard BM, Rodseth RN. What evidence is there for intraoperative predictors of perioperative cardiac outcomes? A systematic review. Perioperative medicine (London, England). 2013;2(1):14. Epub 2014/01/30. doi: 10.1186/2047-0525-2-14. PubMed PMID: 24472327; PMCID: Pmc3964323.
29. Walsh M, Devereaux PJ, Garg AX, Kurz A, Turan A, Rodseth RN, Cywinski J, Thabane L, Sessler DI. Relationship between intraoperative mean arterial pressure and clinical outcomes after noncardiac surgery: toward an empirical definition of hypotension. Anesthesiology. 2013;119(3):507-15. Epub 2013/07/10. doi: 10.1097/ALN.0b013e3182a10e26. PubMed PMID: 23835589.
30. Kwon S, Thompson R, Dellinger P, Yanez D, Farrohki E, Flum D. Importance of perioperative glycemic control in general surgery:

a report from the Surgical Care and Outcomes Assessment Program. Annals of surgery. 2013;257(1):8-14. Epub 2012/12/14. doi: 10.1097/SLA.0b013e31827b6bbc. PubMed PMID: 23235393; PMCID: Pmc4208433.
31. Bratzler DW, Houck PM, Surgical Infection Prevention Guideline Writers W. Antimicrobial prophylaxis for surgery: an advisory statement from the National Surgical Infection Prevention Project. American journal of surgery. 2005;189(4):395-404. doi: 10.1016/j.amjsurg.2005.01.015. PubMed PMID: 15820449.
32. Young PY, Khadaroo RG. Surgical site infections. The Surgical clinics of North America. 2014;94(6):1245-64. Epub 2014/12/03. doi: 10.1016/j.suc.2014.08.008. PubMed PMID: 25440122.
33. Kurz A, Sessler DI, Lenhardt R. Perioperative normothermia to reduce the incidence of surgical-wound infection and shorten hospitalization. Study of Wound Infection and Temperature Group. The New England journal of medicine. 1996;334(19):1209-15. Epub 1996/05/09. doi: 10.1056/nejm199605093341901. PubMed PMID: 8606715.
34. High versus low positive end-expiratory pressure during general anaesthesia for open abdominal surgery (PROVHILO trial): a multicentre randomised controlled trial. The Lancet. 2014;384(9942):495-503. doi: 10.1016/s0140-6736(14)60416-5.
35. Futier E, Constantin JM, Jaber S. Protective lung ventilation in operating room: a systematic review. Minerva anestesiologica. 2014;80(6):726-35. Epub 2013/11/15. PubMed PMID: 24226493.
36. Avidan MS, Jacobsohn E, Glick D, Burnside BA, Zhang L, Villafranca A, Karl L, Kamal S, Torres B, O'Connor M, Evers AS, Gradwohl S, Lin N, Palanca BJ, Mashour GA. Prevention of intraoperative awareness in a high-risk surgical population. The New England journal of medicine. 2011;365(7):591-600. Epub 2011/08/19. doi: 10.1056/NEJMoa1100403. PubMed PMID: 21848460.
37. Mashour GA, Shanks A, Tremper KK, Kheterpal S, Turner CR, Ramachandran SK, Picton P, Schueller C, Morris M, Vandervest JC, Lin N, Avidan MS. Prevention of intraoperative awareness with explicit recall in an unselected surgical population: a randomized comparative effectiveness trial. Anesthesiology. 2012;117(4):717-25. Epub 2012/09/20. doi: 10.1097/ALN.0b013e31826904a6. PubMed PMID: 22990178; PMCID: PMC3447261.
38. Mashour GA, Tremper KK, Avidan MS. Protocol for the "Michigan Awareness Control Study": A prospective, randomized, controlled trial comparing electronic alerts based on bispectral index monitoring or minimum alveolar concentration for the prevention of intraoperative awareness. BMC anesthesiology. 2009;9:7. Epub 2009/11/07. doi: 10.1186/1471-2253-9-7. PubMed PMID: 19891771; PMCID: PMC2777120.
39. Avidan MS, Zhang L, Burnside BA, Finkel KJ, Searleman AC, Selvidge JA, Saager L, Turner MS, Rao S, Bottros M, Hantler C, Jacobsohn E, Evers AS. Anesthesia awareness and the bispectral index. The New England journal of medicine. 2008;358(11):1097-108. Epub 2008/03/14. doi: 10.1056/NEJMoa0707361. PubMed PMID: 18337600.
40. Behrends M, DePalma G, Sands L, Leung J. Association between intraoperative blood transfusions and early postoperative delirium in older adults. Journal of the American Geriatrics Society. 2013;61(3):365-70. Epub 2013/03/19. doi: 10.1111/jgs.12143. PubMed PMID: 23496244.
41. Bernard AC, Davenport DL, Chang PK, Vaughan TB, Zwischenberger JB. Intraoperative transfusion of 1 U to 2 U packed red blood cells is associated with increased 30-day mortality, surgical-site infection, pneumonia, and sepsis in general surgery patients. Journal of the American College of Surgeons. 2009;208(5):931-7, 7.e1-2; discussion 8-9. Epub 2009/05/30. doi: 10.1016/j.jamcollsurg.2008.11.019. PubMed PMID: 19476865.
42. Nair BG, Horibe M, Newman SF, Wu WY, Peterson GN, Schwid HA. Anesthesia information management system-based near real-time decision support to manage intraoperative hypotension and hypertension. Anesthesia and analgesia. 2014;118(1):206-14. Epub 2013/11/20. doi: 10.1213/ane.0000000000000027. PubMed PMID: 24247227.
43. Lipton JA, Barendse RJ, Schinkel AF, Akkerhuis KM, Simoons ML, Sijbrands EJ. Impact of an alerting clinical decision support system for glucose control on protocol compliance and glycemic control in the intensive cardiac care unit. Diabetes Technol Ther. 2011;13(3):343-9. Epub 2011/02/05. doi: 10.1089/dia.2010.0100. PubMed PMID: 21291336.
44. Sathishkumar S, Lai M, Picton P, Kheterpal S, Morris M, Shanks A, Ramachandran SK. Behavioral Modification of Intraoperative Hyperglycemia Management with a Novel Real-time Audiovisual Monitor. Anesthesiology. 2015. Epub 2015/05/23. doi: 10.1097/aln.0000000000000699. PubMed PMID: 26001031.
45. Dawson NV, Arkes HR. Systematic errors in medical decision making: judgment limitations. Journal of general internal medicine. 1987;2(3):183-7. Epub 1987/05/01. PubMed PMID: 3295150.
46. Stiegler MP, Ruskin KJ. Decision-making and safety in anesthesiology. Current opinion in anaesthesiology. 2012;25(6):724-9. Epub 2012/11/07. doi: 10.1097/ACO.0b013e328359307a. PubMed PMID: 23128454.
47. Stiegler MP, Tung A. Cognitive processes in anesthesiology decision making. Anesthesiology. 2014;120(1):204-17. Epub 2013/11/12. doi: 10.1097/aln.0000000000000073. PubMed PMID: 24212195.
48. McDonald CJ. Protocol-based computer reminders, the quality of care and the non-perfectability of man. The New England journal of medicine. 1976;295(24):1351-5. Epub 1976/12/09. doi: 10.1056/nejm197612092952405. PubMed PMID: 988482.
49. Patel VL, Kaufman DR, Arocha JF. Emerging paradigms of cognition in medical decision-making. Journal of biomedical informatics. 2002;35(1):52-75. Epub 2002/11/06. PubMed PMID: 12415726.
50. de Man FR, Greuters S, Boer C, Veerman DP, Loer SA. Intra-operative monitoring--many alarms with minor impact. Anaesthesia. 2013;68(8):804-10. Epub 2013/06/12. doi: 10.1111/anae.12289. PubMed PMID: 23745968.
51. Weinger MB, Reddy SB, Slagle JM. Multiple measures of anesthesia workload during teaching and nonteaching cases. Anesthesia and analgesia. 2004;98(5):1419-25, table of contents. Epub 2004/04/24. PubMed PMID: 15105224.
52. Kruger GH, Tremper KK. Advanced integrated real-time clinical displays. Anesthesiology clinics. 2011;29(3):487-504. Epub 2011/08/30. doi: 10.1016/j.anclin.2011.05.004. PubMed PMID: 21871406.
53. Rothman B, Sandberg WS, St Jacques P. Using information technology to improve quality in the OR. Anesthesiology clinics. 2011;29(1):29-55. Epub 2011/02/08. doi: 10.1016/j.anclin.2010.11.006. PubMed PMID: 21295751.
54. Institute of Medicine Committee on Quality of Health Care in A. Crossing the Quality Chasm: A New Health System for the 21st Century. Washington (DC): National Academies Press (US)
Copyright 2001 by the National Academy of Sciences. All rights reserved.; 2001.
55. Morris AH, Hirshberg E, Sward KA. Computer protocols: how to implement. Best practice & research Clinical anaesthesiology. 2009;23(1):51-67. Epub 2009/05/20. PubMed PMID: 19449616.
56. Egger Halbeis CB, Epstein RH, Macario A, Pearl RG, Grunwald Z. Adoption of anesthesia information management systems by academic departments in the United States. Anesthesia and analgesia. 2008;107(4):1323-9. Epub 2008/09/23. doi: 10.1213/ane.0b013e31818322d2. PubMed PMID: 18806048.
57. Stol IS, Ehrenfeld JM, Epstein RH. Technology diffusion of anesthesia information management systems into academic anesthesia departments in the United States. Anesthesia and analgesia. 2014;118(3):644-50. Epub 2014/02/22. doi: 10.1213/ane.0000000000000055. PubMed PMID: 24557109.
58. Spring SF, Sandberg WS, Anupama S, Walsh JL, Driscoll WD, Raines DE. Automated documentation error detection and notification improves anesthesia billing performance. Anesthesiology. 2007;106(1):157-63. Epub 2007/01/02. PubMed PMID: 17197858.
59. Nair BG, Newman SF, Peterson GN, Wu WY, Schwid HA. Feedback mechanisms including real-time electronic alerts to achieve near 100% timely prophylactic antibiotic administration in surgical cases. Anesthesia and analgesia. 2010;111(5):1293-300. doi: 10.1213/ANE.0b013e3181f46d89. PubMed PMID: 20841414.
60. O'Reilly M, Talsma A, VanRiper S, Kheterpal S, Burney R. An anesthesia information system designed to provide physician-specific feedback improves timely administration of prophylactic antibiotics. Anesthesia and analgesia. 2006;103(4):908-12. Epub

2006/09/27. doi: 10.1213/01.ane.0000237272.77090.a2. PubMed PMID: 17000802.
61. Kooij FO, Klok T, Hollmann MW, Kal JE. Decision support increases guideline adherence for prescribing postoperative nausea and vomiting prophylaxis. Anesthesia and analgesia. 2008;106(3):893-8, table of contents. Epub 2008/02/23. doi: 10.1213/ane.0b013e31816194fb. PubMed PMID: 18292437.
62. Breslow MJ, Rosenfeld BA, Doerfler M, Burke G, Yates G, Stone DJ, Tomaszewicz P, Hochman R, Plocher DW. Effect of a multiple-site intensive care unit telemedicine program on clinical and economic outcomes: an alternative paradigm for intensivist staffing. Critical care medicine. 2004;32(1):31-8. Epub 2004/01/07. doi: 10.1097/01.ccm.0000104204.61296.41. PubMed PMID: 14707557.
63. Nassar BS, Vaughan-Sarrazin MS, Jiang L, Reisinger HS, Bonello R, Cram P. Impact of an intensive care unit telemedicine program on patient outcomes in an integrated health care system. JAMA internal medicine. 2014;174(7):1160-7. Epub 2014/05/14. doi: 10.1001/jamainternmed.2014.1503. PubMed PMID: 24819673.
64. Wilcox ME, Adhikari NK. The effect of telemedicine in critically ill patients: systematic review and meta-analysis. Critical care (London, England). 2012;16(4):R127. Epub 2012/07/20. doi: 10.1186/cc11429. PubMed PMID: 22809335; PMCID: Pmc3580710.
65. Ahmed A, Chandra S, Herasevich V, Gajic O, Pickering BW. The effect of two different electronic health record user interfaces on intensive care provider task load, errors of cognition, and performance. Critical care medicine. 2011;39(7):1626-34. Epub 2011/04/12. doi: 10.1097/CCM.0b013e31821858a0. PubMed PMID: 21478739.
66. Pickering BW, Dong Y, Ahmed A, Giri J, Kilickaya O, Gupta A, Gajic O, Herasevich V. The implementation of clinician designed, human-centered electronic medical record viewer in the intensive care unit: A pilot step-wedge cluster randomized trial. International journal of medical informatics. 2015;84(5):299-307. Epub 2015/02/17. doi: 10.1016/j.ijmedinf.2015.01.017. PubMed PMID: 25683227.
67. Pickering BW, Herasevich V, Ahmed A, Gajic O. Novel Representation of Clinical Information in the ICU: Developing User Interfaces which Reduce Information Overload. Applied clinical informatics. 2010;1(2):116-31. Epub 2010/01/01. doi: 10.4338/aci-2009-12-cr-0027. PubMed PMID: 23616831; PMCID: Pmc3632280.
68. Steurbaut K, Colpaert K, Gadeyne B, Depuydt P, Vosters P, Danneels C, Benoit D, Decruyenaere J, De Turck F. COSARA: integrated service platform for infection surveillance and antibiotic management in the ICU. Journal of medical systems. 2012;36(6):3765-75. Epub 2012/04/25. doi: 10.1007/s10916-012-9849-8. PubMed PMID: 22527780.
69. Steurbaut K, Van Hoecke S, Colpaert K, Lamont K, Taveirne K, Depuydt P, Benoit D, Decruyenaere J, De Turck F. Use of web services for computerized medical decision support, including infection control and antibiotic management, in the intensive care unit. Journal of telemedicine and telecare. 2010;16(1):25-9. Epub 2010/01/21. doi: 10.1258/jtt.2009.001008. PubMed PMID: 20086264.
70. Current record count for the Cochrane Library [cited 2015 February 19]. Available from: http://www.cochranelibrary.com/about/about-the-cochrane-library.html - CENTRAL.
71. Osborne NH, Nicholas LH, Ryan AM, Thumma JR, Dimick JB. Association of hospital participation in a quality reporting program with surgical outcomes and expenditures for Medicare beneficiaries. Jama. 2015;313(5):496-504. Epub 2015/02/04. doi: 10.1001/jama.2015.25. PubMed PMID: 25647205.
72. Etzioni DA, Wasif N, Dueck AC, Cima RR, Hohmann SF, Naessens JM, Mathur AK, Habermann EB. Association of hospital participation in a surgical outcomes monitoring program with inpatient complications and mortality. Jama. 2015;313(5):505-11. Epub 2015/02/04. doi: 10.1001/jama.2015.90. PubMed PMID: 25647206.
73. Balust J, Macario A. Can anesthesia information management systems improve quality in the surgical suite? Current opinion in anaesthesiology. 2009;22(2):215-22. Epub 2009/04/25. doi: 10.1097/ACO.0b013e328324b9e6. PubMed PMID: 19390248.
74. Rosenbloom ST, Geissbuhler AJ, Dupont WD, Giuse DA, Talbert DA, Tierney WM, Plummer WD, Stead WW, Miller RA. Effect of CPOE user interface design on user-initiated access to educational and patient information during clinical care. Journal of the American Medical Informatics Association : JAMIA. 2005;12(4):458-73. Epub 2005/04/02. doi: 10.1197/jamia.M1627. PubMed PMID: 15802487; PMCID: Pmc1174891.
75. Weiss CH, Dibardino D, Rho J, Sung N, Collander B, Wunderink RG. A clinical trial comparing physician prompting with an unprompted automated electronic checklist to reduce empirical antibiotic utilization. Critical care medicine. 2013;41(11):2563-9. Epub 2013/08/14. doi: 10.1097/CCM.0b013e318298291a. PubMed PMID: 23939354; PMCID: Pmc3812385.
76. Weiss CH, Moazed F, McEvoy CA, Singer BD, Szleifer I, Amaral LA, Kwasny M, Watts CM, Persell SD, Baker DW, Sznajder JI, Wunderink RG. Prompting physicians to address a daily checklist and process of care and clinical outcomes: a single-site study. American journal of respiratory and critical care medicine. 2011;184(6):680-6. Epub 2011/05/28. doi: 10.1164/rccm.201101-0037OC. PubMed PMID: 21616996; PMCID: Pmc3208596.
77. Kahn JM, Gunn SR, Lorenz HL, Alvarez J, Angus DC. Impact of nurse-led remote screening and prompting for evidence-based practices in the ICU*. Critical care medicine. 2014;42(4):896-904. Epub 2013/11/10. doi: 10.1097/ccm.0000000000000052. PubMed PMID: 24201176.
78. Ellen ME, Leon G, Bouchard G, Ouimet M, Grimshaw JM, Lavis JN. Barriers, facilitators and views about next steps to implementing supports for evidence-informed decision-making in health systems: a qualitative study. Implementation science : IS. 2014;9(1):179. Epub 2014/12/06. doi: 10.1186/s13012-014-0179-8. PubMed PMID: 25476735; PMCID: Pmc4299810.
79. Damschroder LJ, Aron DC, Keith RE, Kirsh SR, Alexander JA, Lowery JC. Fostering implementation of health services research findings into practice: a consolidated framework for advancing implementation science. Implementation science : IS. 2009;4:50. Epub 2009/08/12. doi: 10.1186/1748-5908-4-50. PubMed PMID: 19664226; PMCID: Pmc2736161.
80. Schubert A, Eckhout GV, Ngo AL, Tremper KK, Peterson MD. Status of the anesthesia workforce in 2011: evolution during the last decade and future outlook. Anesthesia and analgesia. 2012;115(2):407-27. Epub 2012/07/05. doi: 10.1213/ANE.0b013e3182575b4e. PubMed PMID: 22759857.
81. Bainbridge D, Martin J, Arango M, Cheng D. Perioperative and anaesthetic-related mortality in developed and developing countries: a systematic review and meta-analysis. Lancet. 2012;380(9847):1075-81. Epub 2012/09/25. doi: 10.1016/s0140-6736(12)60990-8. PubMed PMID: 22998717.

1.7 Describe EACH of your participant populations

- Include description of any control group(s)
- Specify the Inclusion/Exclusion criteria for EACH group

On a daily basis during the study period (weekdays from 7am to 5pm), each operating room at Barnes-Jewish Hospital where anesthesiology services are provided will be eligible for randomization, which will occur daily at the level of the operating room using computer-generated assignment. The experimental arm will receive the additional support of the Anesthesiology Control Tower in the form of control-tower alerts to the attending anesthesiologist that complement the AlertWatch® system. Some of these alerts will use video conference calling for communication between the clinician in the control tower and the clinicians in the operating room.

1.13 Provide a detailed description in sequential order of the study procedures following the consent process - DO NOT cut and paste from the Consent Document.

Describe study populations separately if they will be participating in different procedures

DESCRIBE:

- Control populations, if applicable
- Any randomization, if applicable
- What participants will be asked to do/what happens in the study (in sequential order)
- The time period over which procedures will occur
- Long-term follow-up and how it occurs

The study is being conducted with a waiver of consent. On a daily basis during the study period (weekdays from 7am to 5pm), each operating room at Barnes-Jewish Hospital where anesthesiology services are provided will be eligible for randomization, which will occur daily at the level of the operating room using computer-generated assignment. The experimental arm will receive the additional support of the Anesthesiology Control Tower in the form of control-tower alerts to the attending anesthesiologist that complement the AlertWatch® system. Some of these alerts will use video conference calling for communication between the clinician in the control tower and the clinicians in the operating room.

1.14 Will participants be randomized?
Yes

1.15 Will any of the following be used to collect information from the participant or others?

- Screening questions or screening/eligibility questionnaires
- Surveys
- Questionnaires
- Stimuli
- Any other written assessments

No

1.16 Does this project involve creating any audio, video, or photographs?
No

1.17 Does the study include any form of deception (e.g., providing participants with false information, misleading information, or withholding information about certain study procedures)?
Examples:

- Procedure includes a cover story that provides a plausible but inaccurate account of the purposes of the research.
- Participants will be provided with false information regarding the particular behaviors of interest in the research.
- Procedures include a confederate pretending to be another participant in the study.
- Participants will be told that the research includes completion of a particular task, when in fact, that task will not be administered.
- Study is designed to introduce a new procedure (or task) that participants are not initially told about.

No

1.18 Indicate any payments or reimbursements to participants (check all that apply)

- None - subjects not paid/reimbursed

1.19 Does this study have a plan to have an individual or committee review combined data from all participants on a periodic basis (such as summary or aggregate safety and/or efficacy data)?
No

1.20 What have you done to minimize any risks?

- No forseeable risks

1.21 What are the potential benefits related to this project for:

- the participant (if any)
- benefits to society (if any)

There is a possible benefit to participants (patients) as they could benefit from extra monitoring from the ACT.

This work could have a major impact on healthcare if it demonstrates that successful implementation of an ACT promotes quality improvement and enhances surgical patient safely, while simultaneously increasing efficiency.

1.22 Provide a summary of the analysis methods you will use, including, if applicable, the data points or outcomes you will analyze.
The baseline data on compliance metrics, surrogate measures of outcomes and clinical outcomes from the 3-month calibration period will be presented using standard descriptive statistics. Once the ACT intervention is implemented, we anticipate a possible contamination (or learning) effect over time in the control group. Clinicians may become sensitized to the standards of practice and the surrogate measures of outcome, leading to “overlapping” improvements in these measures in both study groups over the course of the study. This learning effect might manifest most strongly among physicians who spent time as “controllers” and “deputy controllers” in the ACT. Furthermore, with the knowledge that clinicians are being observed, there is a high likelihood of a robust Hawthorne effect (109, 110). The baseline data will be useful in assessing the extent of these effects. We will analyze results from the interventional period in 3-month time bins (with baseline data for reference) to evaluate the extent, potential increase over time, and potential durability of the contamination and Hawthorne effects. The behaviors and objective physiological variables measured under Specific Aims 2a and 2b might prove useful in quantifying the contamination and Hawthorne effects over time, although these effects could be difficult to distinguish from each other. Comparisons between groups during the randomized pilot study will be with parametric and non-parametric statistical tests, according to the distributions of the measures of interest. Fisher’s exact or Chi-square test will be used (1) to assess whether there is a difference between groups in the proportion of times that administration of antibiotics

is >30 minutes before or after surgical incision; (2) to evaluate whether or not the ACT alerts improve the compliance with 5-minute interval blood pressure measurement and with the use of EEG monitoring when indicated; and (3) to ascertain whether or not the alerts are associated with improved documentation of neuromuscular function. Contingency statistical tests will be used to compare occurrence of hypotension and hypothermia between groups, and unpaired t-test or Mann-Whitney U test, as appropriate, will be used to compare these durations between groups. Similar statistical approaches will be used for the exploratory outcomes, all of which will be regarded as hypothesis generating. ANCOVA or non-parametric ANCOVA might be helpful in detecting (and potentially controlling for) patterns of contamination or a Hawthorne effect (111). The results obtained in this pilot trial will be used to estimate appropriate sample sizes for follow-up outcomes studies. By convention, statistical significance will be based on a two-sided p value <0.05. All statistical testing will be with SAS® version 9.4 (SAS Institute Inc., Cary, North Carolina, USA).

1.23 Provide the rationale or power analysis to support the number of participants proposed to complete this study.
This pilot study will be carried out in all operating rooms at Barnes-Jewish Hospital (BJH, South Campus), St. Louis, MO, where AlertWatch® has been installed since 2014 and the electronic perioperative medical record has been in place since 2009. BJH is a 1,252 bed academic university affiliated and adult tertiary care hospital, performing about 19,000 inpatient surgeries a year (86). On average, 125 surgeries take place every business day of the week. To be conservative, we estimate that information on 50 to 100 surgeries per day will be collected for this pilot study. We therefore anticipate a total sample size of about 3,000 surgeries during the pre-intervention period, and about 21,000 surgeries during the trial period with 10,500 surgeries in each arm. We estimate that we will have comprehensive outcomes data for approximately 8,000 of the 21,000 patients included in the pilot study.

1.24 Indicate below only the sources of your data or specimens that you are requesting to obtain under a WAIVER OF CONSENT:

**Type**	**Source**	**# records/samples**	**Any identifiers (check all that apply)?**
Data - Retrospective<br>Records created between:<br>Earliest: 4/1/16<br>latest: 11/26/19	• Repository/Registry<br>• Research Data Core (RDC) (collection of vital status and date of death)	15158	• Names<br>• Medical record numbers<br>• Account numbers<br>• Any elements of dates (except year) for dates that are directly related to an individual, including birth date, admission date, discharge date, death date, and all ages over 89 and all elements of dates (including year) indicative of such age, except that such ages and elements may be aggregated into a single category of age 90 or older.<br>• A unique identifying number or code that can be linked to identifiers
Data - Retrospective<br>Records created between:<br>Earliest: 4/1/2016<br>latest: 12/14/17	• Medical Records (Metevision, Clinical Desktop, alertwatch, EPIC)<br>• Collected Under an Approved IRB Study (201203088, 201506017, Division of Cardiothoracic Surgery's Cardiac Surgery Quality assurance surgical database)<br>• Other (On a daily basis during the study period (weekdays from 7am to 5pm), each operating room of (BJH) will be eligible for cluster randomization, which will occur at the level of the operating room.)	21000	• Medical record numbers<br>• Account numbers<br>• A unique identifying number or code that can be linked to identifiers<br>• Names<br>• Any elements of dates (except year) for dates that are directly related to an individual, including birth date, admission date, discharge date, death date, and all ages over 89 and all elements of dates (including year) indicative of such age, except that such ages and elements may be aggregated into a single category of age 90 or older.
Data - Prospective/Ongoing	• Medical Records (Metevision, Clinical Desktop, alertwatch, EPIC)<br>• Collected Under an Approved IRB Study (201203088, 201506017, Division of Cardiothoracic Surgery's Cardiac Surgery Quality assurance surgical database)<br>• Other (On a daily basis during the study period (weekdays from 7am to 5pm), each operating room of (BJH) will be	21000	• Medical record numbers<br>• Account numbers<br>• Names<br>• A unique identifying number or code that can be linked to identifiers<br>• Any elements of dates (except year) for dates that are directly related to an individual, including birth date, admission date, discharge date, death date, and all ages over 89 and all elements of dates (including year) indicative of such age, except that such ages and elements may be aggregated into a single category of age 90 or older.

Type	Source	# records/samples	Any identifiers (check all that apply)?
	eligible for cluster randomization, which will occur at the level of the operating room.)		
Data - Prospective/Ongoing	• Repository/Registry<br>• Research Data Core (RDC) (collection of vital status and date of death)	15158	• A unique identifying number or code that can be linked to identifiers<br>• Any elements of dates (except year) for dates that are directly related to an individual, including birth date, admission date, discharge date, death date, and all ages over 89 and all elements of dates (including year) indicative of such age, except that such ages and elements may be aggregated into a single category of age 90 or older.<br>• Medical record numbers<br>• Account numbers

Attachment Name	Category	Version	Date Attached
Variable_Definitions_Targeted_Pancreatectomy_150101.pdf	Listing of Data/Specimen Data Points	1	12/13/17
doc02741120150529095246(1).pdf	Listing of Data/Specimen Data Points	1	12/13/17
Variable_Definitions_Targeted_Thyroidectomy_150101.pdf	Listing of Data/Specimen Data Points	1	12/13/17
Variable_Definitions_Classic_150101.pdf	Listing of Data/Specimen Data Points	1	12/13/17
Variable_Definitions_Targeted_VentralHerniaRepair_150101.pdf	Listing of Data/Specimen Data Points	1	12/13/17
Variable_Definitions_Targeted_Proctectomy_150101.pdf	Listing of Data/Specimen Data Points	1	12/13/17
Variable_Definitions_Targeted_AIEndo_150101.pdf	Listing of Data/Specimen Data Points	1	12/13/17
Variable_Definitions_Targeted_AIOpen_150101.pdf	Listing of Data/Specimen Data Points	1	12/13/17
Variable_Definitions_Targeted_Appendectomy_150101.pdf	Listing of Data/Specimen Data Points	1	12/13/17
Variable_Definitions_Targeted_CarotidEndo_150101.pdf	Listing of Data/Specimen Data Points	1	12/13/17
Variable_Definitions_Targeted_Hepatectomy_150101.pdf	Listing of Data/Specimen Data Points	1	12/13/17
Variable_Definitions_Targeted_LEOpen_150101.pdf	Listing of Data/Specimen Data Points	1	12/13/17
Variable_Definitions_Targeted_LEEndo_150101.pdf	Listing of Data/Specimen Data Points	1	12/13/17
RDC data points 12.6.19.docx	Listing of Data/Specimen Data Points	1	12/06/19
Variable_Definitions_Targeted_CarotidOpen_150101.pdf	Listing of Data/Specimen Data Points	1	12/13/17
Variable_Definitions_Targeted_Colectomy_150101.pdf	Listing of Data/Specimen Data Points	1	12/13/17
WUSM DataCollectionForm v2_81 (custom).docx	Listing of Data/Specimen Data Points	1	12/13/17
Variables List_5.3.18.docx	Listing of Data/Specimen Data Points	3	05/09/18
Chapter 4 Hysterectomy PT 1-1-15(1).pdf	Listing of Data/Specimen Data Points	1	12/13/17

Chapter 4 Reconstruction PT 1-1-15.pdf	Listing of Data/Specimen Data Points	1	12/13/17
Variable_Definitions_Targeted_AAAEndo_150101.pdf	Listing of Data/Specimen Data Points	1	12/13/17
Variable_Definitions_Targeted_AAAOpen_150101.pdf	Listing of Data/Specimen Data Points	1	12/13/17
Worksheet_Targeted_Jan_2015(1).pdf	Listing of Data/Specimen Data Points	1	12/13/17
Data points 201603038.docx	Listing of Data/Specimen Data Points	3	12/14/17

1.24.a Does your data include **protected health information (PHI)**?
Yes

1.24.b What is the plan for participant identifiers?
Identifiers will be destroyed at the earliest opportunity, consistent with the conduct of the research.

1.24.c Does the research team agree that the requested information will not be reused or disclosed to any other person or entity, except as required by law, for authorized oversight of the study, or for other research for which the use or disclosure of the requested information would be permitted by the HIPAA Privacy Rule?
Yes

1.25 Will any data from this project be stored for use in future research studies?
No

1.26 Does this project involve the collection or use of biological samples or genetic data?
No

1.27 Are you requesting institutional certification to contribute human data or samples to a repository or database for broad sharing (public or restricted access)?
No

2. Participants

2.1 Will there be any adult participants?
Yes

2.1.a How many adult participants do you expect to consent or enroll under a waiver for this project?
21000

2.1.b What is the age of the youngest adult participant?
18.0

2.1.c What is the age of the oldest adult participant?
No age limit

2.2 Will there be any minor participants?
No

2.3 Will there be any emancipated minor participants?
No

2.9 Is this project about pregnant women?
No

2.10 Will this project involve fetuses?
No

2.11 Does this project involve the use of fetal tissue from any source?
No

2.13 Does this project involve prisoners as participants?
No

3. Performance Sites

3.1 Indicate type of site(s) where research will occur (check all that apply):
- Hospital

- Academic Institution

3.2 Where will project procedures take place (check all that apply)?

- School of Medicine
- Barnes Jewish Hospital (BJH)

3.3 Is this project also being conducted by other researchers at their own sites (e.g. a multi-site collaborative project)?

No

5. Privacy & Confidentiality

5.1 Indicate your plans to protect the privacy interests of the participants during the conduct of the study (check all that apply):

- Only the minimum necessary private information is collected for the purposes of the study
- Any procedures or interventions conducted as part of the study will be conducted in private setting to the extent possible

5.3 Project uses paper or hard copy consents, surveys, data collection forms, research subject binders, or other hard copy materials (check all that apply):

No

5.4 Project collects, stores and/or transmits electronic data on mobile devices, desktop computers, servers including cloud servers, email, or any other information in electronic form (check all that apply):

Yes

- Password protected
- Access is limited to research team only
- Data are encrypted
- Transmitted using recognized security for electronic submission

5.5 Project collects or uses biologic specimens (check all that apply):

No

5.6 Identify any additional protections in place for data and or samples (check all that apply):

- No additional protections
