# Supplementary material for: An Intraoperative Telemedicine Program to Improve Perioperative Quality Measures: The ACTFAST-3 Randomized Clinical Trial
Source: JAMA Netw Open. 2023 Sep 22;6(9):e2332517. doi: 10.1001/jamanetworkopen.2023.32517 (PMC10517374; doi:10.1001/jamanetworkopen.2023.32517)
Supplement: Supplement 2. — eTable 1. Outcome Definitions eTable 2. ACT Contact Activity eTable 3. Common Alerts eTable 4. Descriptive Statistics Excluding Surgeries Within 30 Days of an Index Operation eTable 5. Treatment Effects Excluding Surgeries Within 30 Days of an Index Operation eTable 6. Descriptive Statistics Stratified by Intervention, Ignoring Prior Treatment Assignments in Subsequent Surgeries eTable 7. Treatment Effects, Ignoring Prior Treatment Assignments in Subsequent Surgeries eFigure 1A. Number of Days per Month With Included Cases (ACT Staffed, Randomization Operational, Outcomes Measured) eFigure 1B. Mean Number of Alerts per Case Over Time eFigure 1C. Number of Contacts per Case Over Time eFigure 2. Secondary and Clinical Outcomes Over Time [file jamanetwopen-e2332517-s002.pdf]

Supplementary Online Content

King CR, Gregory S, Fritz BA, et al; ACTFAST Study Group. Impact of an intraoperative telemedicine program on perioperative quality measures: the ACTFAST-3 randomized clinical trial. *JAMA Netw Open*. 2023;6(9):e2332517. doi:10.1001/jamanetworkopen.2023.32517

eTable 1. Outcome Definitions

eTable 2. ACT Contact Activity

eTable 3. Common Alerts

eTable 4. Descriptive Statistics Excluding Surgeries Within 30 Days of an Index Operation

eTable 5. Treatment Effects Excluding Surgeries Within 30 Days of an Index Operation

eTable 6. Descriptive Statistics Stratified by Intervention, Ignoring Prior Treatment Assignments in Subsequent Surgeries

eTable 7. Treatment Effects, Ignoring Prior Treatment Assignments in Subsequent Surgeries

eFigure 1A. Number of Days per Month With Included Cases (ACT Staffed, Randomization Operational, Outcomes Measured)

eFigure 1B. Mean Number of Alerts per Case Over Time

eFigure 1C. Number of Contacts per Case Over Time

eFigure 2. Secondary and Clinical Outcomes Over Time

This supplemental material has been provided by the authors to give readers additional information about their work.

© 2023 King CR et al. *JAMA Network Open*.

eTable 1. Outcome Definitions

Hypotension	Duration of time with MAP < 60 mmHg
Temperature monitoring	At least 1 documented intraop temperature (among cases > 1 hour)
Antibiotic redosing	All antibiotic doses followed by a redose within recommended intervals while patient is intraoperative (max 3 doses, excluding administrations with the same drug administered pre-procedure > 1 doses):<br>Drug — Interval (hours)<br>ampicillin — 2<br>aztreonam — 4<br>cefazolin — 4<br>cefepime — 4<br>cefoxitin — 2<br>ceftriaxone — 12<br>ciprofloxacin — 8<br>clindamycin — 6<br>ertapenem — 24<br>gentamycin — 8<br>linezolid — 10<br>meropenem — 2<br>metronidazole — 8<br>piperacillin — 2<br>vancomycin — 12
Intraoperative blood glucose management	Failure = no insulin administered for blood glucose greater than 180 mg/dL OR no intraoperative measurement of blood glucose in patients with type 1 diabetes undergoing cases >= 1 hour OR no intraoperative measurement of blood glucose in patients with type 2 diabetes undergoing cases > 2 hours
Neuromuscular monitoring documentation	Proportion of cases with a train of four documented prior to extubation if a nondepolarizing neuromuscular blocking agent was administered
Ventilator management	Among cases with mechanical ventilation, failure = median tidal volume more than 10 mL/kg ideal body mass
Volatile anesthetic utilization	Mean fresh gas flow rates for cases with volatile anesthetic use > 80% of case duration

© 2023 King CR et al. *JAMA Network Open*.

Postoperative acute renal failure	In first 7 postoperative days, KDIGO stage of AKI. Where baseline creatinine unavailable, age, sex, height, race, and weight normed value imputed.
Postoperative atrial fibrillation	Flowsheet records of incident atrial fibrillation in first 7 postop days, atrial fibrillation billing diagnosis not present on admission
Postoperative respiratory failure	Any mechanical ventilation in first 7 postoperative days, excluding patients with preoperative ventilation (Epic era only)
Postoperative delirium	Any positive CAM-ICU score in first 7 postoperative days, excluding patients with preoperative positive CAM-ICU in the prior 7 days.
Intraoperative awareness	Removed from analysis plan – not consistently evaluated
Surgical site infection	Removed from analysis plan due to low data quality. Defined as billing ICD10-CM codes T81.4, K68.11, O86.0, T8[34].5, T8[23].6, T8[245].7 excluding “D” and “S” codes (subsequent encounter and sequelae) as well as present-on-admission.
30-day readmission	Admission to any BJC facility in 30 days after discharge from index procedure
30-day mortality	Death recorded in first 30 postoperative days

© 2023 King CR et al. *JAMA Network Open*.

eTable 2. ACT Contact Activity

Group	Intervention	Control	Intervention	Control
Period	Pre-Epic	Pre-Epic	Epic	Epic
Number of Cases	5808	6065	7172	7209
Number of Alerts	9281	9488	53644	53732
Fraction w alert	0.65	0.65	0.97	0.97
Num w contact	877	40	759	59
Num w alert contact	877	40	521	34
Num w review contact	0	0	367	31
Num contact w significant issue	367	20	370	34
Num contact affecting management	387	20	216	11

© 2023 King CR et al. *JAMA Network Open*.

eTable 3. Common Alerts

MESSAGE	**N**	**PRE-EPIC**	**EPIC**
MAP TRENDING DOWN. POTENTIAL HYPOTENSION.	9100	0	9100
HIGH CUMULATIVE VASOPRESSOR BOLUS DOSE.	8599	4330	4269
TACHYCARDIA. HR =	7121	3209	3912
PONV RISK. CONSIDER TWO OR MORE ANTIEMETICS.	6609	0	6609
NO BP MEASURED	6574	0	6574
PONV RISK. [A] ADMINISTERED. CONSIDER ADDITIONAL	6396	0	6396
HYPOTENSION: MAP =	5899	0	5899
TIDAL VOLUME = [A] ([B] CC/KILO OF IDEAL BODY WEI	5803	1398	4405
LOW TEMPERATURE =	5358	0	5358
CONSIDER DOCUMENTING TRAIN OF FOUR.	4747	3587	1160
TACHYCARDIA. HR =	4444	1950	2494
HYPERTENSION. MAP =	3968	0	3968
HIGH MAC =	3776	0	3776
LOW MAC =	3056	0	3056
ANTIBIOTIC HAS NOT BEEN DOCUMENTED PRIOR TO…	2741	0	2741
CUMULATIVE TIME FOR MAP < [A] = [B] MINUTES.	2718	623	2095
LOW CO2 =	2696	0	2696
LOW MAC =	2517	0	2517
HYPOTENSION: MAP =	2449	0	2449
LOW PEEP =	2446	0	2446

© 2023 King CR et al. *JAMA Network Open*.

eTable 4. Descriptive Statistics Excluding Surgeries Within 30 Days of an Index Operation

Stratified by intervention. P-values for quantitative variables by Mann-Whitney U, factor variables by Fisher's exact test.

	Control	Intervention
N	12457	12179
Age (median [IQR])	60 [47, 69]	60 [47, 69]
Body Mass Index (median [IQR])	29 [24, 34]	29 [24, 34]
Sex n (%)		
Female	6387 (51.3)	6361 (52.2)
Male	6066 (48.7)	5813 (47.7)
Other	4 (0.0)	5 (0.0)
Race n (%)		
White	9585 (76.9)	9371 (76.9)
Black	2516 (20.2)	2438 (20.0)
Other	210 (1.7)	226 (1.9)
Asian	146 (1.2)	144 (1.2)
Surgery Type n (%)		
Other	2072 (16.6)	2038 (16.7)
Orthopedics	1429 (11.5)	1386 (11.4)
Cardiac and Thoracic	1295 (10.4)	1195 (9.8)
Gynecology	1379 (11.1)	1376 (11.3)
Urology	1330 (10.7)	1235 (10.1)
General	852 (6.8)	875 (7.2)
Neurology	852 (6.8)	849 (7.0)
Otolaryngology	838 (6.7)	773 (6.3)
Vascular	741 (5.9)	748 (6.1)
Gastroenterology	390 (3.1)	439 (3.6)
Colorectal	423 (3.4)	414 (3.4)
Transplant	376 (3.0)	354 (2.9)
Hepatobiliary	336 (2.7)	370 (3.0)
Plastics	144 (1.2)	127 (1.0)
ASA n (%)		
1	542 (4.9)	548 (5.0)
2	4491 (40.2)	4370 (39.9)
3	4984 (44.6)	4975 (45.4)
4	1130 (10.1)	1035 (9.4)
5	27 (0.2)	26 (0.2)
Barthel index < 100 n (%)	1350 (10.8)	1406 (11.5)
Coronary Artery Disease n (%)	1525 (13.4)	1464 (13.1)
Congestive Heart Failure n (%)	1158 (10.2)	1117 (10.0)
Atrial Fibrillation n (%)	1024 (9.0)	984 (8.8)
Peripheral Arterial Disease n (%)	911 (8.0)	860 (7.7)

© 2023 King CR et al. *JAMA Network Open*.

Diabetes Mellitus n (%)	2667 (27.6)	2614 (27.6)
Cirrhosis = 1 (%)	232 (2.0)	229 (2.1)
Functional Capacity < 4 METs n (%)	3078 (30.7)	3128 (31.5)
Hypertension n (%)	5960 (52.3)	5824 (52.2)
COPD or Asthma n (%)	2253 (19.8)	2176 (19.5)
End Stage Renal Disease n (%)	516 (4.4)	528 (4.6)
Cerebrovascular Disease n (%)	808 (7.1)	693 (6.2)
Current Cancer n (%)	2508 (22.0)	2477 (22.2)

© 2023 King CR et al. *JAMA Network Open*.

eTable 5. Treatment Effects Excluding Surgeries Within 30 Days of an Index Operation

Coef = rate ratio for binary (n %) and ordinal (median IQR) outcomes, regression coefficient for continuous (mean SD) outcomes. CI = 95% confidence interval by GEE clustered on OR and day. AKI, delirium, respiratory failure, atrial fibrillation exclude patients with those characteristics preoperatively. AKI= acute kidney injury (KDIGO stage), TV = tidal volume. See supplemental table 1 for definitions. P-values Holm corrected in each group of outcomes (2 primary, 8 secondary, 6 clinical tests).

Outcome	Intervention	Control	coef (CI)	p
**Primary outcomes**				
Not postop hypothermic, n (%)	7109/10796 (66)	7261/11009 (66)	1.00 (0.98, 1.02)	1
Not postop hyperglycemic, n (%)	6923/8069 (86)	6989/8169 (86)	1.00 (0.99, 1.02)	1
**Secondary outcomes**				
Glucose measurement done, n (%)	1258/1833 (69)	1189/1853 (64)	1.07 (1.02, 1.12)	0.04
Intra low map minutes, mean (SD)	7 (17)	7 (18)	-0.22 (-0.67, 0.23)	1
Temperature monitoring, n (%)	8185/8731 (94)	8336/8943 (93)	1.01 (1.00, 1.01)	1
No missed antibiotics, n (%)	11971/12179 (98)	12237/12457 (98)	1.00 (1.00, 1.00)	1
Insulin per clinical guidelines, n (%)	609/947 (64)	680/1015 (67)	0.96 (0.90, 1.02)	1
Neuromuscular monitoring documented, n (%)	4471/6263 (71)	4420/6318 (70)	1.02 (1.00, 1.04)	0.665
Appropriate TV, n (%)	4632/4955 (93)	4650/4994 (93)	1.00 (0.99, 1.01)	1
Fresh gas flow L/min, mean (SD)	3 (1)	3 (1)	-0.00 (-0.05, 0.04)	1
**Clinical outcomes**				
AKI stage			0.98 (0.88, 1.10)	1
= 0, n (%)	10780/11479 (94%)	11012/11738 (94%)		
= 1, n (%)	531/11479 (5%)	553/11738 (5%)		
= 2, n (%)	75/11479 (1%)	70/11738 (1%)		
= 3, n (%)	93/11479 (1%)	103/11738 (1%)		
30-day mortality, n (%)	168/12179 (1)	194/12457 (2)	0.89 (0.72, 1.09)	0.996
30-day readmission, n (%)	499/12179 (4)	441/12457 (4)	1.16 (1.02, 1.32)	0.156
Delirium, n (%)	195/546 (36)	187/543 (34)	1.04 (0.88, 1.22)	1
Respiratory failure, n (%)	204/10781 (2)	204/10946 (2)	1.02 (0.83, 1.24)	1
Incident atrial fibrillation, n (%)	268/11053 (2)	307/11291 (3)	0.89 (0.76, 1.05)	0.85
SSI, n/total (%)	284/12179 (2)	306/12457 (2)	0.95 (0.81, 1.12)	0.531
multipleCases, n/total (%)	2336/12179 (19)	2410/12457 (19)	0.99 (0.94, 1.05)	0.752

© 2023 King CR et al. *JAMA Network Open*.

eTable 6. Descriptive Statistics Stratified by Intervention, Ignoring Prior Treatment Assignments in Subsequent Surgeries

P-values for quantitative variables by Mann-Whitney U, factor variables by Fisher's exact test.

	Control	Intervention
N	13289	12965
Age (median [IQR])	60 [47, 69]	60 [47, 69]
Body Mass Index (median [IQR])	28 [24, 34]	29 [24, 34]
Sex n (%)		
Female	6706 (50.5)	6687 (51.6)
Male	6579 (49.5)	6273 (48.4)
Other	4 (0.0)	5 (0.0)
Race n (%)		
White	10204 (76.8)	9965 (76.9)
Black	2714 (20.4)	2613 (20.2)
Other	222 (1.7)	239 (1.8)
Asian	149 (1.1)	148 (1.1)
Surgery Type n (%)		
Other	2146 (16.1)	2104 (16.2)
Orthopedics	1520 (11.4)	1467 (11.3)
Cardiac and Thoracic	1465 (11.0)	1359 (10.5)
Gynecology	1391 (10.5)	1396 (10.8)
Urology	1397 (10.5)	1292 (10.0)
General	985 (7.4)	985 (7.6)
Neurology	894 (6.7)	907 (7.0)
Otolaryngology	893 (6.7)	814 (6.3)
Vascular	795 (6.0)	800 (6.2)
Gastroenterology	455 (3.4)	517 (4.0)
Colorectal	440 (3.3)	426 (3.3)
Transplant	392 (2.9)	364 (2.8)
Hepatobiliary	342 (2.6)	379 (2.9)
Plastics	174 (1.3)	155 (1.2)
ASA n (%)		
1	552 (4.6)	556 (4.8)
2	4685 (39.2)	4522 (38.7)
3	5380 (45.0)	5377 (46.0)
4	1303 (10.9)	1197 (10.2)
5	33 (0.3)	30 (0.3)
Barthel index < 100 n (%)	1417 (10.7)	1476 (11.4)
Coronary Artery Disease n (%)	1652 (13.6)	1595 (13.4)
Congestive Heart Failure n (%)	1301 (10.7)	1244 (10.5)
Atrial Fibrillation n (%)	1143 (9.4)	1109 (9.3)
Peripheral Arterial Disease n (%)	1021 (8.4)	962 (8.1)

© 2023 King CR et al. *JAMA Network Open*.

Diabetes Mellitus n (%)	2889 (28.0)	2844 (28.2)
Cirrhosis (%)	267 (2.2)	251 (2.1)
Functional Capacity < 4 METs n (%)	3247 (30.3)	3308 (31.3)
Hypertension n (%)	6413 (52.7)	6244 (52.5)
COPD or Asthma n (%)	2456 (20.2)	2353 (19.8)
End Stage Renal Disease n (%)	569 (4.5)	571 (4.7)
Cerebrovascular Disease n (%)	885 (7.3)	763 (6.4)
Current Cancer n (%)	2627 (21.6)	2597 (21.8)

© 2023 King CR et al. *JAMA Network Open*.

eTable 7. Treatment Effects, Ignoring Prior Treatment Assignments in Subsequent Surgeries

Coef = rate ratio for binary (n %) and ordinal (median IQR) outcomes, regression coefficient for continuous (mean SD) outcomes. CI = 95% confidence interval by GEE clustered on OR and day. AKI, delirium, respiratory failure, atrial fibrillation exclude patients with those characteristics preoperatively. AKI= acute kidney injury (KDIGO stage), TV = tidal volume. See supplemental table 1 for definitions. P-values Holm corrected in each group of outcomes.

Outcome	Intervention	Control	coef (CI)	p
**Primary outcomes**				
Not postop hypothermic, n (%)	7600/11433 (66)	7785/11686 (67)	1.00 (0.98, 1.02)	1
Not postop hyperglycemic, n (%)	7469/8693 (86)	7535/8798 (86)	1.00 (0.99, 1.02)	1
**Secondary outcomes**				
Glucose measurement done, n (%)	1355/1977 (69)	1265/1981 (64)	1.07 (1.03, 1.12)	0.016
Intra low map minutes, mean (SD)	7 (17)	7 (18)	-0.22 (-0.66, 0.21)	1
Temperature monitoring, n (%)	8637/9219 (94)	8792/9452 (93)	1.01 (1.00, 1.02)	0.833
No missed antibiotics, n (%)	12738/12965 (98)	13036/13289 (98)	1.00 (1.00, 1.00)	1
Insulin per clinical guidelines, n (%)	648/1013 (64)	717/1073 (67)	0.96 (0.90, 1.02)	1
Neuromuscular monitoring documented, n (%)	4621/6577 (70)	4609/6670 (69)	1.02 (0.99, 1.04)	1
Appropriate TV, n (%)	5036/5388 (93)	5083/5452 (93)	1.00 (0.99, 1.01)	1
Fresh gas flow L/min, mean (SD)	3 (1)	3 (1)	-0.01 (-0.05, 0.03)	1
**Clinical outcomes**				
AKI stage			0.99 (0.89, 1.10)	1
= 0, n (%)	11438/12177 (94%)	11685/12448 (94%)		
= 1, n (%)	561/12177 (5%)	575/12448 (5%)		
= 2, n (%)	77/12177 (1%)	74/12448 (1%)		
= 3, n (%)	101/12177 (1%)	114/12448 (1%)		
30-day mortality, n (%)	206/12965 (2)	232/13289 (2)	0.91 (0.76, 1.10)	1
30-day readmission, n (%)	568/12965 (4)	526/13289 (4)	1.11 (0.98, 1.25)	0.576
Delirium, n (%)	224/637 (35)	210/622 (34)	1.04 (0.89, 1.22)	1
Respiratory failure, n (%)	236/11289 (2)	234/11493 (2)	1.03 (0.85, 1.23)	1
Incident atrial fibrillation, n (%)	306/11673 (3)	340/11968 (3)	0.92 (0.79, 1.08)	1

© 2023 King CR et al. *JAMA Network Open*.

eFigure 1A. Number of Days per Month With Included Cases (ACT Staffed, Randomization Operational, Outcomes Measured)

Circle = actual value, x = number of weekdays. No adjustment for holidays.

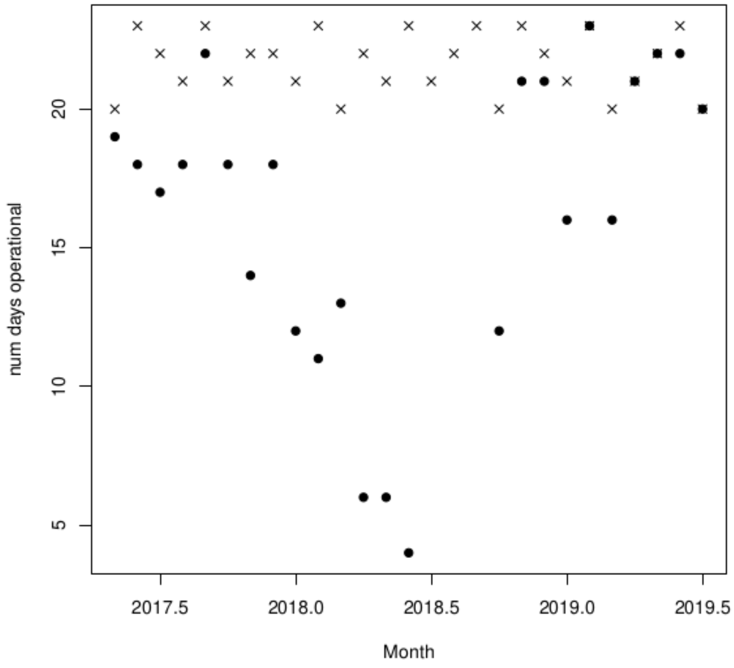

© 2023 King CR et al. *JAMA Network Open*.

eFigure 1B. Mean Number of Alerts per Case Over Time

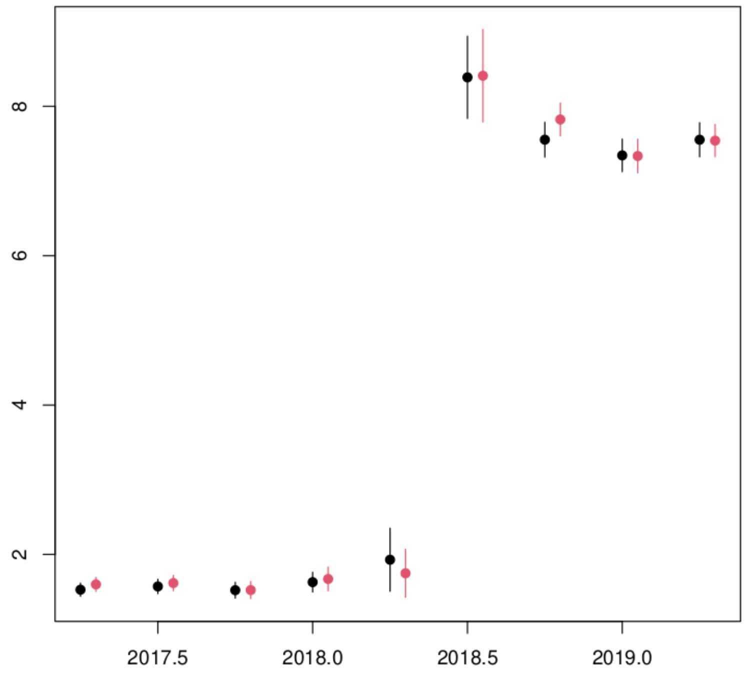

© 2023 King CR et al. *JAMA Network Open*.

eFigure 1C. Number of Contacts per Case Over Time

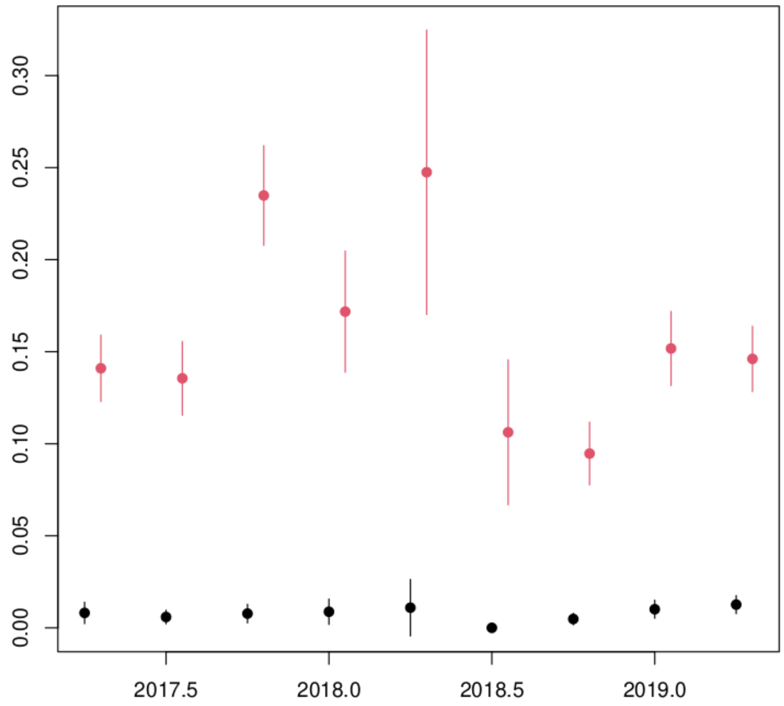

© 2023 King CR et al. *JAMA Network Open*.

eFigure 2. Secondary and Clinical Outcomes Over Time

Dotted line = EHR transition. AKI_st2 = KDIGO grade 2 or higher AKI, outcomes defined in Sup Table 1. Confidence intervals by clustered GEE with linear response on proportion or outcome.

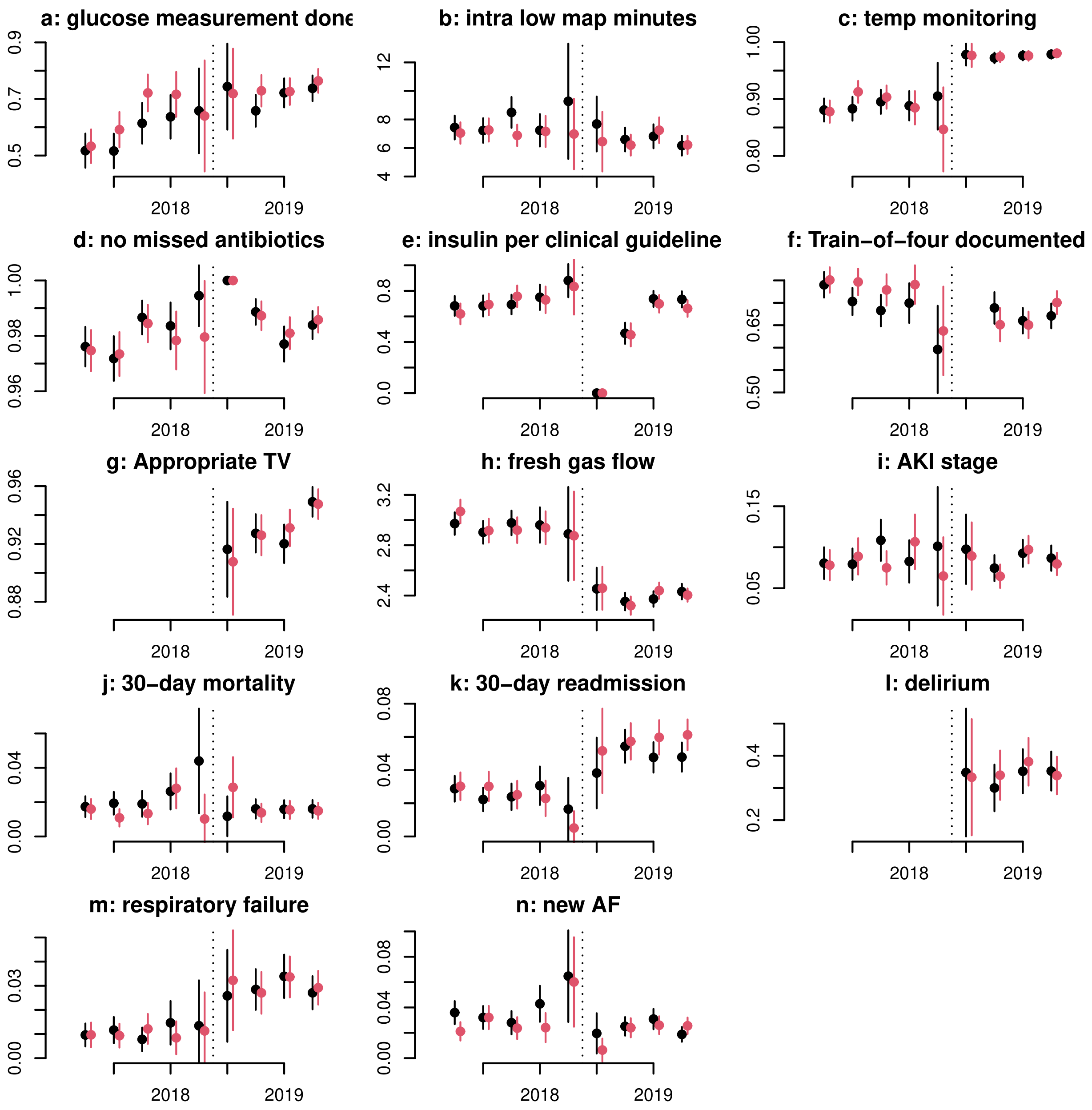

© 2023 King CR et al. *JAMA Network Open*.
