# Supplementary material for: An Intraoperative Telemedicine Program to Improve Perioperative Quality Measures: The ACTFAST-3 Randomized Clinical Trial
Source: JAMA Netw Open. 2023 Sep 22;6(9):e2332517. doi: 10.1001/jamanetworkopen.2023.32517 (PMC10517374; doi:10.1001/jamanetworkopen.2023.32517)
Supplement: Supplement 3. — Nonauthor Collaborators. Members of the ACTFAST Study Group [file jamanetwopen-e2332517-s003.pdf]

Supplemental Online Content: Nonauthor Collaborators
*First name, last name, and suffix (if applicable) are required and will appear in PubMed.

*Group Name(s): Members of the ACTFAST Study Group							
***First Name and Middle Initial(s)**	***Last Name**	***Suffix (eg, Jr, III)**	Academic Degrees	Institution	Location (city, state/province, country)	Role or Contribution, eg, chair, principal investigator	Group (if more than 1 Group listed in the byline) and/or Subgroup (eg, Steering Committee)
Mohamed	Abdelhack		PhD	WUSTL		Infrastructure team	
Amrita	Aranake-Chrisinger		MD	WUSTL		ACT Clinician	
Aaron	Archer		MSN	WUSTL		ACT Clinician	
Maureen	Arends			WUSTL		Administrative and coordination team	
Emily	Armstrong		BSN	WUSTL		ACT Clinician	
Umeshkumar	Athiraman		MD	WUSTL		ACT Clinician	
Sennaraj	Balasubramanian		MD	WUSTL		ACT Clinician	
Anchal	Bansal		BS	WUSTL		ACT Clinician	
Kara	Battig		BSN	WUSTL		ACT Clinician	
Danielle	Benematti		BSN	WUSTL		ACT Clinician	
George	Benzinger	III	MD, PHD	WUSTL		ACT Clinician	
Mara	Bollini		BA, BSN, MHA	WUSTL		ACT Clinician	
Anuradha	Borle		MD	WUSTL		ACT Clinician	
Michael	Bottros		MD, MD	WUSTL		ACT Clinician	
Walter	Boyle		MD	WUSTL		ACT Clinician	
Thomas	Bozada		BSN, MS	WUSTL		ACT Clinician	
Margaret	Bradley		BSN	WUSTL		ACT Clinician	
BrandonUfert	BrandonUfert		RN	WUSTL		ACT Clinician	
Christina	Brown		MD	WUSTL		ACT Clinician	
Jamie	Brown-Shpigel		MD	WUSTL		ACT Clinician	
Jamila	Burton		BS	WUSTL		ACT Clinician	
Megan	Carmony		MD	WUSTL		ACT Clinician	
Kathryn	Cass		CRNA	WUSTL		ACT Clinician	
Laura	Cavallone		MD	WUSTL		ACT Clinician	

Supplemental Online Content: Nonauthor Collaborators
*First name, last name, and suffix (if applicable) are required and will appear in PubMed.

***First Name and Middle Initial(s)**	***Last Name**	***Suffix (eg, Jr, III)**	Academic Degrees	Institution	Location (city, state/province, country)	Role or Contribution, eg, chair, principal investigator	Group (if more than 1 Group listed in the byline) and/or Subgroup (eg, Steering Committee)
Yunwei	Chen		MD	WUSTL		ACT Clinician	
Yixin	Chen		PHD	WUSTL		Infrastructure team	
Han	Choi		MD	WUSTL		ACT Clinician	
Marissa	Coggin		DNP	WUSTL		ACT Clinician	
Zachary	Cohen		BSE, MD	WUSTL		ACT Clinician	
Casey	Critchlow			WUSTL		Administrative and coordination team	
Christopher	Davies		MD	WUSTL		ACT Clinician	
Christopher	Davis		MD	WUSTL		ACT Clinician	
Aaron	Demler-Barth		BSN	WUSTL		ACT Clinician	
Ryan	Durk		MD	WUSTL		ACT Clinician	
Daniel	Eddins		BSN	WUSTL		ACT Clinician	
David	Eisenbath		BSN	WUSTL		ACT Clinician	
Meredith	Ellis		RN	WUSTL		ACT Clinician	
Daniel	Emmert		MD	WUSTL		ACT Clinician	
Krisztina	Escallier		MD	WUSTL		ACT Clinician	
Jane	Exler		BS	WUSTL		ACT Clinician	
Mitchell	Fingerman		MD	WUSTL		ACT Clinician	
Ellen	Fischbach		CCRP	WUSTL		Administrative and coordination team	
Elizabeth	Frasca		BSN	WUSTL		ACT Clinician	
Michelle	Ge		MD	WUSTL		ACT Clinician	
Jason	Gillihan		MD	WUSTL		ACT Clinician	
Marie	Goez		DNP	WUSTL		ACT Clinician	
Natasha	Goodwin		BSN	WUSTL		ACT Clinician	
Thomas	Graetz		MD	WUSTL		ACT Clinician	
Ryan	Guffey		MD	WUSTL		ACT Clinician	
Shelly	Gupta		BA	WUSTL		ACT Clinician	
Katharine	Gurba		MD	WUSTL		ACT Clinician	
Kelsey	Gutesa		BSN	WUSTL		ACT Clinician	

Supplemental Online Content: Nonauthor Collaborators
*First name, last name, and suffix (if applicable) are required and will appear in PubMed.

***First Name and Middle Initial(s)**	***Last Name**	***Suffix (eg, Jr, III)**	Academic Degrees	Institution	Location (city, state/province, country)	Role or Contribution, eg, chair, principal investigator	Group (if more than 1 Group listed in the byline) and/or Subgroup (eg, Steering Committee)
Tracey	Guthrie		BSN, CCRC	WUSTL		ACT Clinician	
Michael	Hakim		MD	WUSTL		ACT Clinician	
Charles	Hantler		MD	WUSTL		ACT Clinician	
Peter	Haw		BSN	WUSTL		ACT Clinician	
Hilary	Heeger		DNP	WUSTL		ACT Clinician	
Erin	Herrera		MSN	WUSTL		ACT Clinician	
Alex	Hincker		MD	WUSTL		ACT Clinician	
Robert	Hovis		CRNA	WUSTL		ACT Clinician	
Gary	Hubbard		MS	WUSTL		ACT Clinician	
Rocco	Hueneke		MD	WUSTL		ACT Clinician	
Mark	Ingram		MSN	WUSTL		ACT Clinician	
Zahid	Iqbal		MD	WUSTL		ACT Clinician	
Susan	Ironstone		MD	WUSTL		ACT Clinician	
Kyle	Jacobsen		MD	WUSTL		ACT Clinician	
Nisha	Jain		MD	WUSTL		ACT Clinician	
Bassel	Kadi		MD	WUSTL		ACT Clinician	
Ivan	Kangrga		MD	WUSTL		ACT Clinician	
Menelaos	Karanikolas		MD	WUSTL		ACT Clinician	
Bridget	Kinealy		BSN	WUSTL		ACT Clinician	
Mary	Kinworthy		BSN	WUSTL		ACT Clinician	
Holly	Kirkpatrick		BSN	WUSTL		ACT Clinician	
Esad	Kiveric		MD	WUSTL		ACT Clinician	
Andrea	Knibb		BSN	WUSTL		ACT Clinician	
Justin	Knittel		MD	WUSTL		ACT Clinician	
Andreas	Kokefer		MD	WUSTL		ACT Clinician	
Helga	Koman		MD	WUSTL		ACT Clinician	
Joseph	Kras		DDS, MD	WUSTL		ACT Clinician	
Kristin	Kraus		RN	WUSTL		ACT Clinician	
Anand	Lakshminarasimhachar		MD, MD	WUSTL		ACT Clinician	

Supplemental Online Content: Nonauthor Collaborators
*First name, last name, and suffix (if applicable) are required and will appear in PubMed.

***First Name and Middle Initial(s)**	***Last Name**	***Suffix (eg, Jr, III)**	Academic Degrees	Institution	Location (city, state/province, country)	Role or Contribution, eg, chair, principal investigator	Group (if more than 1 Group listed in the byline) and/or Subgroup (eg, Steering Committee)
Joseph	Larese		MD	WUSTL		ACT Clinician	
Chakrapol	Lattanand		MD	WUSTL		ACT Clinician	
Aparna	Malshet Casarella		Bs	WUSTL		ACT Clinician	
Jonathan	Mathew		MD	WUSTL		ACT Clinician	
Hannah	Maybrier		BS	WUSTL		Administrative and coordination team	
Peter	McAvity		MS	WUSTL		ACT Clinician	
Alyssa	McClellan		BSN	WUSTL		ACT Clinician	
Jacob	McDowell		MD	WUSTL		ACT Clinician	
Heather	McKenzie		MD	WUSTL		ACT Clinician	
Alicia	Meng			WUSTL		Study Coordinator	
Angela	Mickle		MS	WUSTL		ACT Clinician	
Melissa	Milbrandt			WUSTL		Administrative and coordination team	
Preet	Mohinder Singh		MD	WUSTL		ACT Clinician	
Alexander	Mohrmann		BSN	WUSTL		ACT Clinician	
David	Monks		MBChB	WUSTL		ACT Clinician	
Arianna	Montes de Oca		MD	WUSTL		Study Coordinator	
Teresa	Murray-Torres		MD	WUSTL		ACT Clinician	
Khatera	Najrabi		BSN	WUSTL		ACT Clinician	
Lauren	Nowakowski			WUSTL		Research assistant	
Ifeanyi	Nwokeabia		MD	WUSTL		ACT Clinician	
Jordan	Oberhaus		BA	WUSTL		ACT Clinician	
Ekua	Owusu-Bediako		MD	WUSTL		ACT Clinician	
Daniel	Park		BA	WUSTL		ACT Clinician	
Aamil	Patel			WUSTL		Research assistant	
Sarah	Perez		CRNA	WUSTL		ACT Clinician	

*First name, last name, and suffix (if applicable) are required and will appear in PubMed.

***First Name and Middle Initial(s)**	***Last Name**	***Suffix (eg, Jr, III)**	Academic Degrees	Institution	Location (city, state/province, country)	Role or Contribution, eg, chair, principal investigator	Group (if more than 1 Group listed in the byline) and/or Subgroup (eg, Steering Committee)
Caroline	Phillips		MA	WUSTL		ACT Clinician	
David	Potter		BSN, MBA	WUSTL		ACT Clinician	
Debra	Pulley		MD	WUSTL		ACT Clinician	
Govind	Rangrass		MD	WUSTL		ACT Clinician	
Janavi	Rao		MD	WUSTL		ACT Clinician	
Rashmi	Rathor		MD	WUSTL		ACT Clinician	
Isabella	Riordan		MD	WUSTL		ACT Clinician	
Isabella	Riordan		MD	WUSTL		ACT Clinician	
Cameron	Ritter		MS, BSN	WUSTL		ACT Clinician	
Evan	Roller		MD	WUSTL		ACT Clinician	
Martha	Sabino			WUSTL		ACT Clinician	
Matthew	Sanzalone		MD	WUSTL		ACT Clinician	
Elvira	Sayfutdinova		BSN	WUSTL		ACT Clinician	
Craig	Schadler		BSN	WUSTL		ACT Clinician	
Elizabeth	Schappe		MS	WUSTL		ACT Clinician	
Alexandra	Schatz		MSN	WUSTL		ACT Clinician	
Anne	Sebastiani		MD	WUSTL		ACT Clinician	
Kimberly	Seiber		MSN	WUSTL		ACT Clinician	
Anshuman	Sharma		MD	WUSTL		ACT Clinician	
Sarah	Sillery		MSN	WUSTL		ACT Clinician	
Kate	Silver		MSN	WUSTL		ACT Clinician	
Susan	Siraco		BA	WUSTL		ACT Clinician	
Melanie	Somercik		MSN	WUSTL		ACT Clinician	
Pratyush	Sontha		BA	WUSTL		ACT Clinician	
James	Spencer		BS	WUSTL		ACT Clinician	
Erika	Spencer		MS	WUSTL		ACT Clinician	
Tracey	Stevens		MD	WUSTL		ACT Clinician	
Jasmin	Swaniker		MD	WUSTL		ACT Clinician	
Martha	Szabo		MD	WUSTL		ACT Clinician	
Raghu	Terkonda		MD	WUSTL		ACT Clinician	

Supplemental Online Content: Nonauthor Collaborators
*First name, last name, and suffix (if applicable) are required and will appear in PubMed.

***First Name and Middle Initial(s)**	***Last Name**	***Suffix (eg, Jr, III)**	Academic Degrees	Institution	Location (city, state/province, country)	Role or Contribution, eg, chair, principal investigator	Group (if more than 1 Group listed in the byline) and/or Subgroup (eg, Steering Committee)
Carolyn	Thai		MD	WUSTL		ACT Clinician	
Marko	Todorovic		MD	WUSTL		ACT Clinician	
Brian	Tolly		MD	WUSTL		ACT Clinician	
Emma	Trammel		BS	WUSTL		ACT Clinician	
Sandhya	Tripathi		PhD	WUSTL		Infrastructure Team	
Lisa	Tseng		MD	WUSTL		ACT Clinician	
Bradley	Uding		BS	WUSTL		ACT Clinician	
Ravi	Upadhyayula		BS	WUSTL		ACT Clinician	
Swarup	Varaday		MBBS,FRCA(UK),FCARSI(IRELAND), MD	WUSTL		ACT Clinician	
William	Varnum		MSN	WUSTL		ACT Clinician	
Minna	Wang		MD	WUSTL		ACT Clinician	
Sarah	Watkins		BSN	WUSTL		ACT Clinician	
Mark	Willingham		BS	WUSTL		ACT Clinician	
William	Wise		MSN	WUSTL		ACT Clinician	
Rachel	Wolfe		PharmD	WUSTL		Protocol development	
Maxim	Wolfson		BS	WUSTL		ACT Clinician	
Hannah	Woodstock		BSN	WUSTL		ACT Clinician	
Randy	Wright		BA	WUSTL		ACT Clinician	
Katie	Yang		MD	WUSTL		ACT Clinician	
Branden	Yee		BA, MD, MPH	WUSTL		ACT Clinician	
Jennifer	Yuan		MD	WUSTL		ACT Clinician	
Paul	Zanaboni		PHD, MD, PhD	WUSTL		ACT Clinician	
Jessica	Zenga		MD	WUSTL		ACT Clinician	

Supplemental Online Content: Nonauthor Collaborators
*First name, last name, and suffix (if applicable) are required and will appear in PubMed.

***First Name and Middle Initial(s)**	***Last Name**	***Suffix (eg, Jr, III)**	Academic Degrees	Institution	Location (city, state/province, country)	Role or Contribution, eg, chair, principal investigator	Group (if more than 1 Group listed in the byline) and/or Subgroup (eg, Steering Committee)
James	Zhang		MD	WUSTL		ACT Clinician	
Tianyang	Zheng		BS	WUSTL		Research assistant	
Nicholas	Zimick		MD	WUSTL		ACT Clinician	
Joshua	Zwingelberg		MD	WUSTL		ACT Clinician	
