# Supplementary material for: An Intraoperative Telemedicine Program to Improve Perioperative Quality Measures: The ACTFAST-3 Randomized Clinical Trial
Source: JAMA Netw Open. 2023 Sep 22;6(9):e2332517. doi: 10.1001/jamanetworkopen.2023.32517 (PMC10517374; doi:10.1001/jamanetworkopen.2023.32517)
Supplement: Supplement 4. — Data Sharing Statement [file jamanetwopen-e2332517-s004.pdf]

Data Sharing Statement

King. Effect of an Intraoperative Telemedicine Program on Perioperative Quality Measures. *JAMA Netw Open*. Published September 22, 2023. doi:10.1001/jamanetworkopen.2023.32517

Data
Data available: No

Additional Information
Explanation for why data not available: Because the primary analysis depends on dates finer than 1 year, it is not HIPAA de-identified. Code and aggregated data will be shared.
